# Chronic endoplasmic reticulum stress in myotonic dystrophy type 2 promotes autoimmunity via mitochondrial DNA release

**DOI:** 10.1038/s41467-024-45535-1

**Published:** 2024-02-20

**Authors:** Sarah Rösing, Fabian Ullrich, Susann Meisterfeld, Franziska Schmidt, Laura Mlitzko, Marijana Croon, Ryan G Nattrass, Nadia Eberl, Julia Mahlberg, Martin Schlee, Anja Wieland, Philipp Simon, Daniel Hilbig, Ulrike Reuner, Alexander Rapp, Julia Bremser, Peter Mirtschink, Stephan Drukewitz, Thomas Zillinger, Stefan Beissert, Katrin Paeschke, Gunther Hartmann, Aleksandra Trifunovic, Eva Bartok, Claudia Günther

**Affiliations:** 1 Department of Dermatology, University Hospital Carl Gustav Carus, TU Dresden, 01307 Dresden, Germany; 2 Institute of Clinical Chemistry and Clinical Pharmacology, University Hospital Bonn, 53127 Bonn, Germany; 3 Institute of Experimental Haematology and Transfusion Medicine, University Hospital Bonn, 53127 Bonn, Germany; 4 Institute for Mitochondrial Diseases and Aging, Faculty of Medicine, CECAD Research Center, 50931 Cologne, Germany; 5 Department of Oncology, Hematology, Rheumatology and Immune-Oncology, University Hospital Bonn, 53127 Bonn, Germany; 6 Department of Neurology, University Hospital Carl Gustav Carus, TU Dresden, 01307 Dresden, Germany; 7 Department of Biology, Cell biology and Epigenetic, Technical University of Darmstadt, Darmstadt, Germany; 8 Institute for Clinical Chemistry and Laboratory Medicine, Faculty of Medicine, TU Dresden, 01307 Dresden, Germany; 9 Core Unit for Molecular Tumor Diagnostics (CMTD), National Center for Tumor Diseases (NCT), Partner Site Dresden, Institute of Human Genetics, University of Leipzig Medical Center, Leipzig, Germany; 10 Unit of Experimental Immunology, Department of Biomedical Sciences, Institute of Tropical Medicine, Antwerp, Belgium

**Keywords:** Autoimmunity, Innate immunity, Skin diseases

## Abstract

Myotonic dystrophy type 2 (DM2) is a tetranucleotide CCTG repeat expansion disease associated with an increased prevalence of autoimmunity. Here, we identified an elevated type I interferon (IFN) signature in peripheral blood mononuclear cells and primary fibroblasts of DM2 patients as a trigger of chronic immune stimulation. Although RNA-repeat accumulation was prevalent in the cytosol of DM2-patient fibroblasts, type-I IFN release did not depend on innate RNA immune sensors but rather the DNA sensor cGAS and the prevalence of mitochondrial DNA (mtDNA) in the cytoplasm. Sublethal mtDNA release was promoted by a chronic activation of the ATF6 branch of the unfolded protein response (UPR) in reaction to RNA-repeat accumulation and non-AUG translated tetrapeptide expansion proteins. ATF6-dependent mtDNA release and resulting cGAS/STING activation could also be recapitulated in human THP-1 monocytes exposed to chronic endoplasmic reticulum (ER) stress. Altogether, our study demonstrates a novel mechanism by which large repeat expansions cause chronic endoplasmic reticulum stress and associated mtDNA leakage. This mtDNA is, in turn, sensed by the cGAS/STING pathway and induces a type-I IFN response predisposing to autoimmunity. Elucidating this pathway reveals new potential therapeutic targets for autoimmune disorders associated with repeat expansion diseases.

## Abstract (web summary)

Myotonic dystrophy type 2 is characterized by large CCUG repeats in the CNBP gene that result in myopathy. Here, the authors show that recognition of aberrant protein translation derived from these repeats leads to ER stress and mitochondrial DNA release, leading to cGAS/STING activation and type-I IFN responses.

## Introduction

Myotonic dystrophy or dystrophia myotonica (DM) is the most common form of muscular dystrophy and characterized by autosomal dominant myopathy with myotonia, progressive muscle weakness and multiorgan involvement^1^. Both major types of DM are caused by tri- or tetranucleotide repeats in DNA^2^. Early-onset myotonic dystrophy type 1 (DM1) is induced by expanded CTG repeats (> 50 repeats) within the 3′ untranslated region of the dystrophia myotonica protein kinase gene^2^. At the mRNA level, expanded noncoding CUG repeats result in the formation of stem-loop dsRNA structures that bind and sequester the RNA-binding proteins, muscleblind-like 1 (MBNL1) and CUG-binding protein 1 (CUGBP1), preventing their normal function as antagonistic regulators of alternative splicing^3^. MBNL1 sequestration results in the expression of its fetal isoform in adult muscle, leading to inappropriate splicing and muscle dysfunction^4,5^.

In contrast, symptoms of DM type 2 (DM2) usually begin in the second to sixth decade and include proximal muscle weakness, grip myotonia, muscle pain and cataracts^1^. Myotonic dystrophy type 2 is induced by expanded CCTG repeats (> 75−11,000 repeats) in intron 1 of cellular nucleic acid-binding protein (CNBP) gene (previously known as zinc finger 9 gene, ZNF9)^6^. In normal individuals, the size of the CCTG repeats in this region is below 30^1^. In DM2, these repeats may expand over the patient’s lifetime but usually contract from one generation to the next, which might explain the late onset of the disease and the lack of a congenital form^1^. CNBP is an RNA-binding protein that binds G-rich elements in target mRNA coding sequences and supports translation by resolving stable mRNA secondary structures^7–9^.

Among the complex organ manifestations in DM2, Tieleman et al. described an enhanced frequency of autoimmune diseases and autoantibodies compared with healthy controls and patients with DM1^10^. Increased incidence of autoimmune phenomena in DM2 patients currently lacks a mechanistic explanation. However, the extended RNA expansions in DM2 compared to DM1^11^ suggest a direct role for the unrestricted nucleic-acid accumulation in its pathogenesis. It has been shown that CUG and CCUG mRNA repeats can form stable, toxic base-paired hairpin structures that translocate from the nucleus to the cytoplasm^12–15^. Such disturbances in nucleic acid metabolism have been linked to autoimmune diseases mediated by the induction of type I interferons (IFN)^16^. Prominent examples include hypomorphic variants of DNase or RNase genes, such as the DNase TREX1 or the SKIV2L RNA exosome, which lead to accumulation of nucleic acids in the cytoplasm and the activation of innate immune receptors that induce IFN and pro-inflammatory cytokine release^17^. While activation of the cytosolic dsDNA sensor cGAMP-synthase (cGAS) / stimulator of interferon genes (STING) pathway is central to IFN-driven disease resulting from DNA accumulation, a number of possible innate immune RNA sensors are potentially downstream of accumulated, aberrantly-structured RNA, including TLR3, TLR7, TLR8, PKR, RIG-I and MDA5^18^.

In the present study, we show that patients with DM2 have a significantly enhanced risk for the development of autoimmune diseases associated with an enhanced type-I IFN-stimulated gene (ISG) signature in blood and tissue. Surprisingly, while we did not detect evidence of direct sensing of expanded RNA repeats by innate RNA sensors in patient cells, we instead observed that RNA and protein repeat expansions induced a cGAS/STING-dependent ISG signature. Although the expanded RNA repeats could not directly activate cGAS, their presence was associated with chronically enhanced endoplasmic reticulum (ER) stress via the ATF6 pathway. In THP-1 monocytes, we could recapitulate this ATF6-dependent activation of cGAS/STING by chronic ER stress and, furthermore, demonstrate that it critically required mitochondrial DNA (mtDNA). Correspondingly, we could also observe mitochondrial stress and mtDNA leakage into the cytoplasm of fibroblasts from DM2 patients. Altogether, our study provides a mechanistic rationale for autoimmune disease in DM2 by linking RNA repeat expansion with ER mitochondrial stress, cGAS activation and the induction of systemic autoinflammation and autoimmunity.

## Results

### Enhanced prevalence of autoimmunity in patients with DM2

**Fig. 1:**
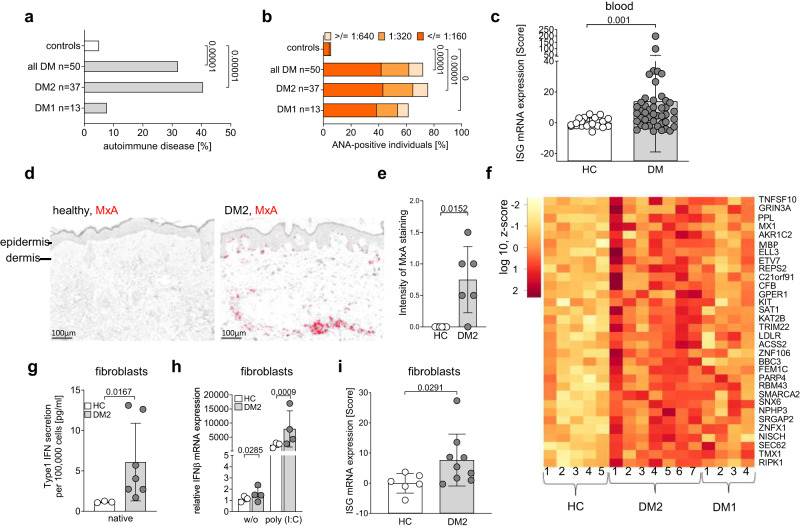
Enhanced type I IFN activation and autoimmunity in patients with myotonic dystrophy. **a** frequency of autoimmune diseases in 50 patients with DM (DM1 *n* = 13, DM2 *n* = 37) compared to the general population^19^ (**b**), antinuclear antibodies (ANA) were determined on Hep-2 cells in the serum of DM1 (*n* = 13) and DM2 (*n* = 37) patients compared with data from a control population (*n* = 1000) measured in the same laboratory^20^. Shown is the percentage of ANA-positive DM1 and DM2 patients (**c**), calculated IFN score^22^ from blood of healthy controls (*n* = 21), DM2 (*n* = 36) and DM1 (*n* = 9) patients. The IFN score was determined using the ISGs IFIT1, IFI44, IFI44L, CXCL10, ISG15, IFI27, and Viperin. **d** representative images of immunohistochemistry of myxovirus resistance protein A (MxA = red) in 4% formaldehyde-fixed lesional skin sections from a healthy control and a DM2 patient. **e** quantification of MxA staining in healthy (*n* = 6) and DM2 (*n* = 6) skin sections. **f** heatmap of ISGs that are significantly increased in 7 DM2 and 4 DM1 fibroblast cell lines compared to 5 control fibroblasts. Each column represents one cell line. The heatmap depicts log 10 values of z-score. **g** determination of type-I IFN expression in the supernatant of healthy control (HC *n* = 3) and DM2 (*n* = 7) fibroblast cell lines. **h** fibroblasts were treated with 10 µg/ml polyinosinic:polycytidylic acid (Poly I:C), and relative mRNA expression of IFNβ in healthy control (*n* = 3) and DM2 (*n* = 4) fibroblasts was measured. Relative expression (n-fold) was calculated to the mean of native healthy controls (*n* = 3). **i** calculated IFN score^22^ from healthy control (HC *n* = 6) and DM2 (*n* = 9) fibroblasts using mRNA expression of the ISGs IFI44, IFI27, ISG15, Viperin, IFI16, IRF7, TLR3. Data are shown as mean ± SD (**c**, **e**, **g**, **h**, **i**). Include data from one (**e**), six (**g**), eleven (**i**) or twelve (**h**) independent experiments. Statistical significance was assessed using Fisher exact test (**a**, **b**), Mann-Whitney U (**c**, **e**, **g**), one-tailed Mann-Whitney U (**h**) or one-tailed student’s *t* test (**i**).

Driven by the observation of cutaneous autoimmune diseases in patients with DM2, we systematically screened 37 patients with DM2, which revealed an enhanced frequency of autoimmune diseases (40.5%) among these patients compared with the overall prevalence of autoimmune diseases in 5−10% of the general population^19^ (Fig. 1a). Autoimmune phenomena covered a wide spectrum including morphea, vitiligo, alopecia areata, sicca syndrome, Raynaud’s syndrome, rheumatoid arthritis, systemic sclerosis, and type I diabetes (Supplementary Table 1 and 2, Supplementary Fig. 1a). Furthermore, the frequency of antinuclear antibodies was significantly enhanced in patients with DM2 (75.7%) and in patients with DM1 (61.5%) compared with a cohort of healthy controls (*n* = 1000)^20^ (Fig. 1b). Disease-specific antibodies directed against SSA(Ro), centromere, SM/RNP and mitochondrial antigens (AMA) were detected (Supplementary Table 3).

### ISG signature in patients with myotonic dystrophy type 2

Chronically sustained and dysregulated type I interferon upregulation has emerged as a main trigger and perpetuator of autoimmune diseases^21^. This cytokine family is an essential mediator of antiviral host defense shifting the innate and adaptive immune response towards inflammation. In line with this, we detected an enhanced type I IFN-stimulated gene (ISG) signature in the blood of DM1 and DM2 patients, as calculated by an IFN score^22^ of 7 different genes (Fig. 1c). Elevated protein levels of the ISG myxovirus resistance protein A (MxA) were detected in morphea lesions of a patient with DM2 (Fig. 1d, Supplementary Fig. 1a) as well as other lesional and non-lesional skin biopsies from DM2 patients (Fig. 1e). For further analysis, we obtained fibroblasts from skin biopsies of 7 patients with DM2, 4 patients with DM1 and 5 healthy controls for RNA sequencing. Among the 313 genes upregulated in DM2 patients (Supplementary data 1), we detected 32 ISGs (Fig. 1f, Supplementary Fig. 1b, c). ISG expression was higher in fibroblasts of patients with DM2 compared with DM1 (Fig. 1f, two-way ANOVA: *p* = 0.0004). Together with the higher prevalence of autoimmunity in DM2 patients (Fig. 1a), this demonstrates both a higher prevalence of type-I IFN stimulation and autoimmunity in DM2. This finding is supported by a previous report by ref. ^10^. which also observed autoimmune diseases predominantly in DM2 when compared to DM1 and to the general population. In addition, a recent analysis in 131 DM2 patients also found a 30% prevalence of autoimmune diseases compared to 5−10% in the general population^19,23^.Therefore, we concentrated on DM2 to attempt to characterize the pathogenic mechanisms leading to ISG upregulation and autoimmunity in these patients. Analysis of IFNβsecretion in fibroblasts from 7 of 9 DM2 patients revealed that patient cells maintained an elevated IFN expression in culture that was not detected in the healthy controls (Fig. 1g). This chronic type I IFN priming led to elevated IFNβ expression before and after stimulation with poly (I:C) in 4 of 9 DM2 patients. (Fig. 1h). In addition to IFNβ, we also detected an enhanced expression of ISGs (IFI44, IFI27, ISG15, Viperin, IFI16, IRF7, TLR3) in cultured DM2 fibroblasts compared to healthy controls (Fig. 1i).

### Cytosolic RNA repeat accumulation in DM2 fibroblasts

**Fig. 2:**
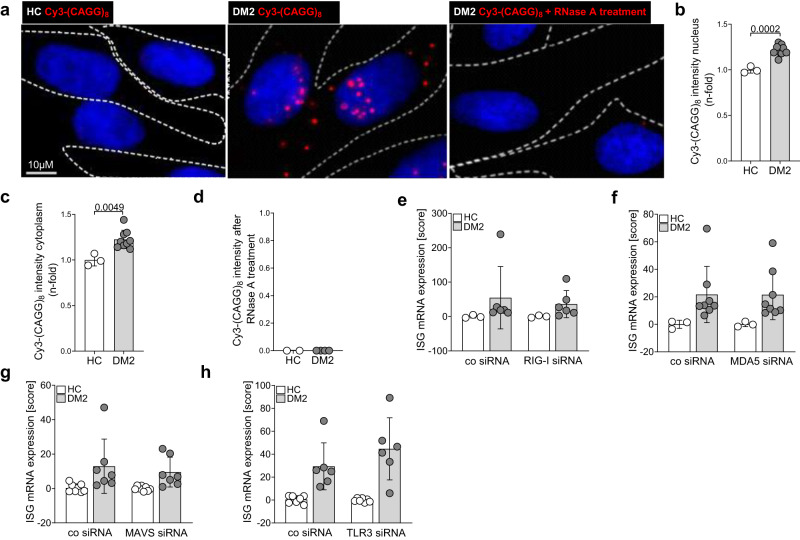
Accumulation of RNA-repeats in the nucleus and the cytoplasm in DM2 fibroblasts. **a** Fluorescence In Situ Hybridization (FISH) of RNA-repeats in fibroblasts. Shown is a representative labeling with the CAGG probe (red) and nuclear staining by DAPI (blue) in one control and one DM2 patient. Pretreatment with 0.5 mg/ml RNase A completely resolved the staining. **b** integrated intensity of RNA-FISH staining in the nucleus (**b**) and cytoplasm (**c**) of healthy controls (*n* = 3) and DM2 patients (*n* = 9). **d** quantification of RNA-FISH staining after treatment of fibroblasts from healthy controls (*n* = 2) and DM2 patients (*n* = 4) with RNase A. Calculated IFN score^22^ using mRNA expression of 4 ISGs (IFI44, IFI27, IRF7, Viperin) after siRNA knockdown of RIG-I (HC *n* = 3, DM2 *n* = 6) (**e**), MDA5 (HC *n* = 3, DM2 *n* = 8) (**f**), MAVS (HC *n* = 8, DM2 *n* = 7) (**g**) or TLR3 (HC *n* = 7, DM2 *n* = 6) (**h**). Data are shown as mean ± SD (**b**–**h**). a-d are representative of three independent experiments. Include data from three (**e**, **f**), four (**h**) or six (**g**) independent experiments. Statistical significance was assessed using student’s *t* test (**b**, **c**).

Improper restriction and compartmentalization of nucleic acids acts as a danger signal for the innate immune system and induces type-I IFN signaling^18^. To investigate whether RNA repeats accumulate in the fibroblasts of DM2 patients and in which subcellular compartment(s), we performed RNA fluorescence in situ hybridization. Using a CAGG fluorescently labeled probe for detection of CCUG repeats, we detected accumulation of repeat RNA in the nucleus as well as the cytoplasm of DM2 fibroblasts, which could be eliminated by RNase treatment and was undetectable in healthy controls (Fig. 2a). Quantification of RNA-FISH staining also confirmed that RNA repeats accumulate not only in the nucleus but also in the cytoplasm (Fig. 2b, c). The repeats were not detected after RNase treatment (Fig. 2d).

### CNBP expression and function is not impaired in DM2 fibroblasts

Repeat expansion in DM2 affects the first intron of the CNBP gene. In DM2 patient fibroblasts, we saw increased mRNA expression of CNBP, but protein levels were in the normal range (Supplementary Fig. 2a, b). Since CNBP is involved in the resolution of stable secondary mRNA and DNA structures and binds to G-rich elements^7,8^, we compared the number of such G-quadruplex structures in patient cells with healthy controls. G-quadruplex levels were slightly elevated in nuclei of DM2 fibroblasts, which may result from the repeat expansions (Supplementary Fig. 2c, d). However, in general, less G-quadruplex structures were detected in the cytoplasm, and there was no difference in G-quadruplex levels between patients and controls (Supplementary Fig. 2e). These findings suggest that G-quadruplex structures in repeat-RNAs might be continuously controlled in both patients and healthy individuals. G-quadruplex unwinding is mainly done by helicases, in particular ATP-dependent RNA helicase DHX36^7^. To investigate if DHX36 is differently expressed in DM2 patients, we analyzed DHX36 expression levels by RT-PCR. Interestingly, mRNA expression of DHX36 was increased, correlating with the upregulation of CNBP mRNA levels (Supplementary Fig. 2f, g). However, CNBP and DHX36 protein levels were in the normal range (Supplementary Fig. 2h).

### Cytosolic RNA repeats are not recognized by RNA sensors

Cytoplasmic RNA with specific structures or modifications can be sensed by the innate immune system^24^. The cytosolic sensors retinoic acid inducible gene I (RIG-I) and melanoma differentiation-associated protein 5 (MDA5) recognize short and phosphorylated (5ʹppp or 5ʹpp blunt, base-paired RNA ≥ 19 bp) or long double-stranded (ds)RNA ( > 300 bp) respectively^24^. Both receptors are broadly expressed and utilize mitochondrial antiviral-signaling protein (MAVS) for their downstream signaling^24^. Toll like receptors (TLR) 3, 7 and 8 recognize RNA in the endosome. While the expression of Toll-like receptor (TLR) 7 and TLR8 is restricted to specific immune cell subsets^18^, and there are no reports of their expression in fibroblasts, TLR3 is expressed on the surface and endosome of fibroblasts^24^ and could potentially sense RNA released from dying cells into cell culture medium.

To analyze whether RIGI, MDA5, MAVS and TLR3 might contribute to type I IFN induction in patient fibroblasts, we downregulated their expression using siRNA and determined the effect on ISG expression. Although siRNA transfection reduced the levels of all RNA receptors targeted (Supplementary Fig. 2i−m), we did not observe changes in expression levels of ISGs (Fig. 2e–h, Supplementary Fig. 2n). To further investigate potential MDA5 activation, we also isolated whole RNA from patient cells and control cells and transfected it into MDA5-expressing and non-MDA5-expressing Hela cells. Although the positive control poly I:C induced a specific response in MDA5-transfected cells, we did not observe upregulation of CXCL10 after transfection of patient RNA (Supplementary Fig. 2o). Moreover, cytosolic transfection of DM2 patient-derived RNA into THP-1 dual sensor cells did not induce activation of an ISRE reporter by IRF3 activation (Supplementary Fig. 2p), although these cells can respond to RIG-I, MDA5, TLR7 and TLR8 activation. Altogether, our data demonstrate that there is no relevant recognition of RNA repeats by cytosolic and endosomal RNA sensors and no indication for direct RNA sensing as the driver of the type-I IFN response in DM2 fibroblasts.

Another sensor for long dsRNA (> 33 base pairs [bp]) or stretches of dsRNA is dsRNA-activated protein kinase (PKR), an RNA restriction factor which upon RNA binding undergoes autophosphorylation and phosphorylates its substrate *translation initiation factor* (eIF2α) at serine 51^25,26^, thereby inhibiting translation initiation and halting protein synthesis^25^. Immunoblot analysis of DM2 patient fibroblasts did not show increased phosphorylation of PKR, indicating that RNA repeats are also unlikely to activate the PKR pathway (Supplementary Fig. 2q).

### RAN translation in DM2

**Fig. 3:**
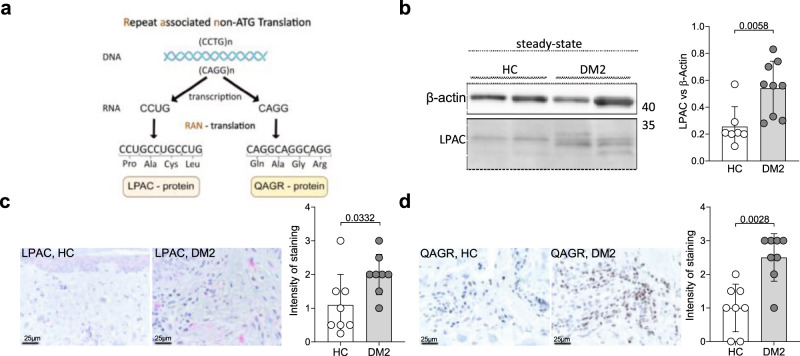
RAN translation in DM2 fibroblasts. **a** schematic representation of the repeat associated non-ATG (RAN) translation. **b** protein concentration of LPAC in DM2 (*n* = 9) and control (*n* = 7) fibroblasts was determined by immunoblotting. **c**, **d** immunohistochemistry (IHC) showing representative LPAC (red) and QAGR (brown) immunostaining in skin section in healthy control and DM2, as well as quantification of staining in healthy controls (*n* = 8) and DM2 patient (*n* = 7) samples. b is representative for 9 DM2 patients. **c**, **d** include data from one independent experiment. Data are shown as mean ± SD. Statistical significance was assessed using student’s *t* test (**c**) or Mann-Whitney U test (**b**, **d**).

Unrestricted RNA repeats can cause a cellular stress response and can be transcribed by repeat-associated non-AUG (RAN) translation. It has been reported that in DM2, CCTG and CAGG expansion mutation are bidirectionally transcribed, and the resulting RNAs are RAN translated, producing tetrapeptide expansion proteins with Leu-Pro-Ala-Cys (LPAC) from the sense strand or Gln-Ala-Gly-Arg (QAGR) repeats from the antisense strand^27^ (Fig. 3a). These proteins were previously shown to accumulate in DM2 patient brains^27^. Analysis of DM2 fibroblasts revealed accumulation of LPAC proteins in patient fibroblasts by Immunoblot (Fig. 3b). The sensitivity of the method was not sufficient to quantify QAGR proteins in fibroblasts. However, we detected elevated protein levels of QAGR as well as LPAC in skin biopsies from DM2 patients with myotonic dystrophy using previously described and validated antibodies^27^. (Fig. 3c, d). To investigate possible cellular consequences of accumulating RAN proteins and RNA repeats, we next analyzed the cellular stress response.

### Chronic activation of the ER stress response in DM2 fibroblasts

**Fig. 4:**
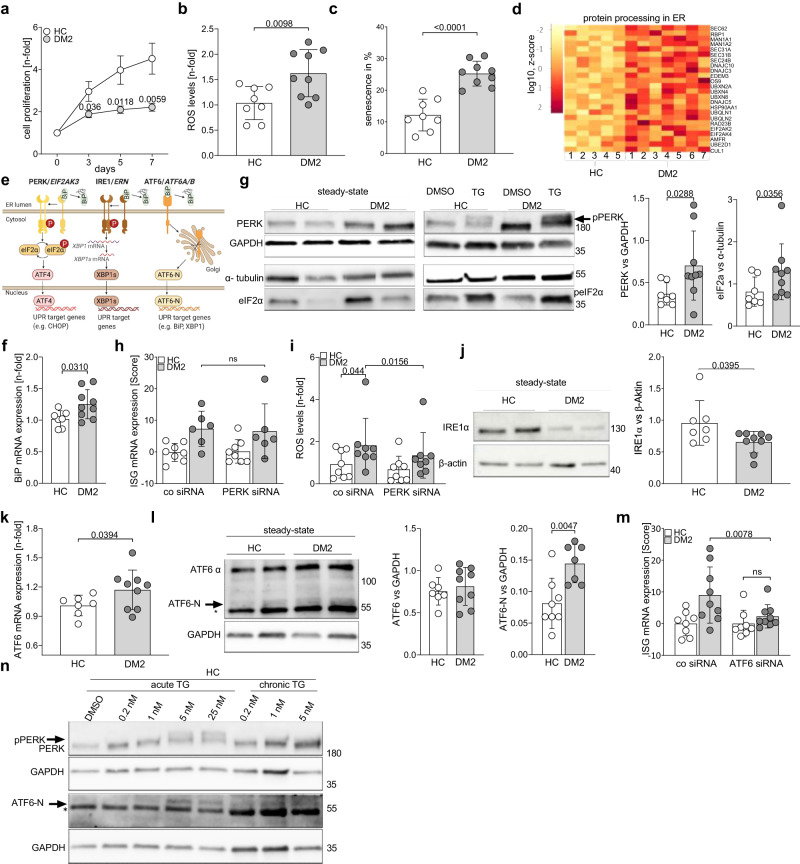
Chronic activation of the ER Stress response in DM2 fibroblasts. In healthy control (HC) or DM2 patient (DM2) fibroblasts, (**a**) cellular proliferation over 7 days, (**b**) ROS levels, and (**c**) the proportion of senescent cells were measured. **d** Heatmap of upregulated genes from the KEGG pathway “Protein processing in ER” from RNAseq (Fig.1). **e** schematic representation of the unfolded protein response (UPR). **f**−**m** depict data from HC and DM2 fibroblasts as indicated. **f** Relative BiP mRNA expression; (**g**) immunoblots of PERK and eIF2α (left) and quantification of all samples (right, HC *n* = 7, DM2 *n* = 9); calculated IFN score^22^ (DM2 *n* = 6, HC *n* = 8) (**h**) and ROS levels (DM2 *n* = 8, HC *n* = 8) (**i**) in PERK siRNA-knockdown fibroblasts; **j** immunoblot of IRE1α (left) and quantification of all samples (right); **k** relative mRNA expression of ATF6. **l** immunoblot of ATF6 and ATF6N (left, and quantification of samples (right)) (ATF6-N DM2: *n* = 7, HC: *n* = 8) (* denotes a non-specific band). **m** calculated IFN score^22^ in ATF6 siRNA-knockdown fibroblasts. **n** depicts HC fibroblasts under conditions of acute (6 h) and chronic (1 week) ER stress, induced by the concentrations of thapsigargin (TG) indicated. Immunoblotting of PERK and ATF6-N levels. **f**, **h**, **k**, **m** mRNA expression was determined using RT-PCR. Include data from three (**a−c**, **f**, **i**, **k**, **m**) or two (**g**, **h**, **j**, **l**) independent experiments. n is representative for 4 healthy donors. **a**−**c**, **m** show data from *n* = 8 healthy controls and *n* = 9 DM2 patient fibroblast lines and **g**, **f**, **j**, **k**, **i** show data from *n* = 7 healthy controls and *n* = 9 DM2 patient fibroblasts lines. a, data are shown as mean ± SEM. **b**, **c**, **f**−**m**, data are shown as mean ± SD. Statistical significance was assessed using student’s *t* test (**a−c**, **f**−**h**, **j**−**l**), one-tailed student’s *t* test (**g**), paired student’s *t* test (**h**), Wilcoxon test (**i**, **m**) or Mann-Whitney *U* test (**a**, **g**, **i**, **m**).

During cell culture, we observed that patient fibroblasts proliferated significantly more slowly compared to the fibroblasts of healthy controls (Fig. 4a) and exhibited a stronger formation of reactive oxygen species (ROS), indicative of cellular stress (Fig. 4b). In addition, we detected an enhanced senescence rate in DM2 fibroblasts by beta-galactosidase staining compared with age-matched controls (Fig. 4c). This enhanced senescence rate is most likely an adaptive response to cellular stress.

Further, unbiased analysis of RNAseq data revealed the induction of genes involved in protein processing in the ER in DM2 fibroblasts compared to both healthy controls and DM1 patients (Supplementary Fig. 3a–c), indicating a stress response (Fig. 4d). ER stress has been previously described as a consequence of the accumulation of RNA repeats and nonsense proteins^28,29^ and results in the UPR signaling (Fig. 4e), which aims to overcome disturbances in cellular homeostasis. The UPR involves three main signaling pathways, each of which is activated by a different ER-sessile sensor of ER stress, i.e., pancreatic ER kinase (PKR)-like ER kinase (PERK), inositol requiring enzyme 1 (IRE1), and activating transcription factor 6 (ATF6)^30^. The sensors remain in an inactive conformation enforced by the direct binding of the chaperone 78 kDa glucose-regulated protein (GRP78 or BiP) to their luminal domains under homeostatic conditions. If BiP is released due to binding of accumulating unfolded or misfolded proteins, the UPR pathways are activated^30^ (Fig. 4e).

In patient fibroblasts, we detected increased mRNA expression of BiP (Fig. 4f), which is in line with the previously reported increase of BiP mRNA in DM1 muscle fibres^31^ and further models of ER stress induced by misfolded proteins^32^. Further analysis demonstrated enhanced protein expression of PERK and its target eIF2α. However, there was no phosphorylation of PERK or eIF2α under steady state conditions, although this could be induced in fibroblasts by thapsigargin (TG), an ER stress inductor used as a positive control (Fig. 4g). Since PERK levels were raised in DM2 fibroblasts, we used siRNA to knockdown this gene in DM2 and healthy control fibroblasts (Supplementary Fig. 3d). While a knockdown of PERK did not decrease ISG mRNA expression (Fig. 4h), it did ameliorate the increased ROS levels in DM2 fibroblasts (Fig. 4i). This connection of PERK with ROS induction is in line with a previous study demonstrating that PERK is required at ER-mitochondrial contact sites to convey apoptosis after ROS-based ER stress^33^.

Moreover, patient fibroblasts also demonstrated a reduced expression of IRE1α protein (Fig. 4j) and did not show an increase in splicing of the IRE1α target XBP1 (Supplementary Fig. 3e), indicating that the IRE1α pathway was not activated in DM2 fibroblasts. In contrast, we found increased expression of ATF6 mRNA in patients compared with healthy controls (Fig. 4k). Moreover, while protein expression of full-length ATF6 was in the range of healthy control fibroblasts, we detected significantly higher levels of N-terminal cleaved ATF6 in patient fibroblasts (Fig. 4l) The specificity of the antibody was demonstrated by time dependent TG induction (Supplementary Fig. 3f). In addition, we found an induction of previously proven ATF6 target genes^34^ (Supplementary Fig. 3g), together indicating activation of the ATF6-mediated ER stress pathway. ATF6 target genes from the GOBP (gene ontology biological process) database were not significantly enriched. To determine the influence of ATF6 signaling on the patients’ fibroblasts, siRNA-mediated downregulation of ATF6 was performed (Supplementary Fig. 3h). Strikingly, depletion of ATF6 reduced the ISG mRNA levels in DM2 fibroblasts to the level of the healthy controls (Fig. 4m), suggesting that the ATF6 pathway is required for ISG upregulation in DM2 fibroblasts. ATF6 signaling has been proposed to mediate cellular adaptation to chronic ER stress^35,36^. Moreover, it has been reported to both drive BiP expression^35,36^ and limit levels of IRE1a expression and activation^35,37^, in line with our observations in DM2 fibroblasts.

We could also recapitulate these findings using low-dose TG (0.2 nM, 1 nM, 5 nM) to mimic chronic ER stress in healthy control fibroblasts over one week. Chronic ER stress induced BiP mRNA expression as did acute ER stress (Supplementary Fig. 3i). In contrast, XBP1 splicing was strongly activated after acute ER stress but not detectable after chronic stress (Supplementary Fig. 3j). Chronic ER stress also did not induce phosphorylation of PERK (Fig. 4n). However, ATF6 mRNA expression was increased by chronic ER stress, and ATF6 protein was cleaved in response to chronic TG stimulation in fibroblasts (Supplementary Fig. 3k, Fig. 4n). These findings confirmed the pattern of chronic ER stress observed in DM2 patient fibroblasts and link chronic ER stress and ATF6 activation to ISG induction in DM2 patient cells.

### Activation of ATF6 mediates ER-mitochondrial crosstalk and cGAS dependent ISG induction

**Fig. 5:**
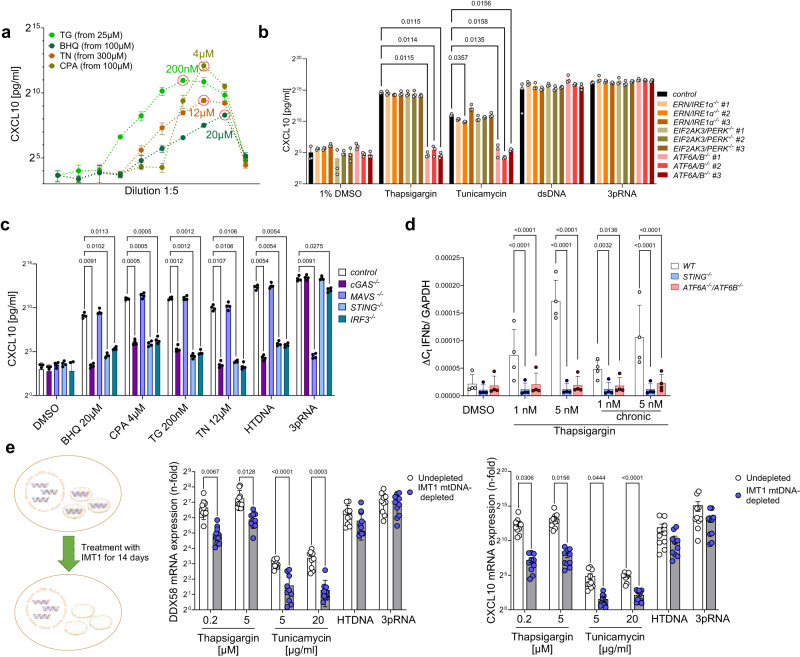
ER stress leads to ATF6 mediated ISG upregulation that depends on sensing of DNA from mitochondria via the cGAS-STING-pathway. **a**−**c** THP-1 cells of the indicated genotype were stimulated with the ER stress inducers cyclopiazonic acid (CPA), 2,5-di-*t*-butyl-1,4-benzohydroquinone (BHQ), thapsigargin (TG) or tunicamycin (TN) at the indicated concentrations or with herring testis (HT)-DNA or 3pRNA. Wildtype THP-1 cells were used in (**a**) and as a “control” in (**b**) and (**c**). Supernatants were harvested 24 h after simulation and probed for CXCL10 levels using ELISA. In (**a**), optimal concentrations for CXCL10 release are circled. **d** relative mRNA expression of IFNB in STING^-/-^ and ATF6^-/-^ THP-1 cells after acute and chronic ER stress induction. For acute ER stress, THP-1 cells were treated once with 1 nM and 5 nM TG. For chronic ER stress, THP-1 cells were treated with 1 nM and 5 nM TG for one week. **e** THP-1 cells were treated with IMT1 for 14 days to deplete mtDNA. Cells were then stimulated as indicated, and CXCL10 and DDX58 mRNA expression were determined by RT-PCR. **a**−**e** data are shown as mean ± SD. Include data from three (**a**, **b**), four (**c**, **d**) or ten (**e**) independent experiments. Statistical significance was assessed using two-way ANOVA and the Bonferroni post hoc test.

To further investigate the signaling pathways potentially leading to ISG induction downstream of ER stress, we used a monocytic cell line, THP-1, which possesses intact pathways for most known RNA sensors and for which CRISPR/Cas9-genome editing is well established^38^. Initially, we tested whether typical inductors of ER stress led to type-I IFN induction in these cells, including the N-glycosylation inhibitor tunicamycin (TN), the SERCA inhibitors TG, cyclopiazonic acid (CPA) and 2,5-di-*t*-butyl-1,4-benzohydroquinone (BHQ) (Fig. 5a). Induction of the ISG CXCL10 could be observed at the protein level for all compounds, albeit at different optimal concentrations. For further experiments, we used representative concentrations of each compound, for which CXCL10 induction (Fig. 5a) was observed.

We then generated THP-1 cells deficient in PERK, ATF6A/B and IRE1α using CRISPR/Cas9 genome editing (Supplementary Table 4) to determine the ER pathway that is responsible for ISG induction in this model. Here, as well, we could clearly observe that deficiency in IRE1α or PERK did not affect ISG induction, while ATF6A/B deficiency completely ablated the ISG response downstream of ER stress (Fig. 5b), confirming the finding of ATF6-dependent ISG induction in DM2 fibroblasts. We then investigated which upstream sensor might be responsible for ISG induction, using THP-1 deficient in cGAS, STING, MAVS and IRF3. For all four activators of ER stress, CXCL10 induction was dependent on the DNA sensor cGAS, its downstream adaptor STING and the transcription factor IRF3, but not MAVS, linking DNA sensing with IFN induction downstream of acute ER stress (Fig. 5c). We then investigated the effect of ATF6 and STING deficiency on IFNβ resulting from chronic ER stress, induced by low-dose TG. Here, as well, STING and ATF6 were critically required for IFNβ induction (Fig. 5d). To investigate non-myeloid cells, we performed the experiments in the epithelial colon carcinoma cell line HT-29. Although these cells were not amenable to experiments with chronic ER stress, under acute ER stress conditions, they also demonstrated cGAS and STING-dependent CXCL10 induction (Supplementary Fig. 4a).

Since ER stress has been associated with release of mtDNA^39^, a potential cGAS ligand, albeit in conjunction with activation of the NLRP3 inflammasome^39^, we then treated THP-1 cells with IMT1 (inhibitor of mitochondrial RNA polymerase essential for mtDNA transcription and replication)^40^ or ethidium bromide (EtBr)^41^ to deplete their mtDNA (Supplementary Fig. 4b, c). IMT1-treatment significantly reduced mtDNA in THP-1 and, strikingly, also specifically blunted the type I IFN response to ER stress indicated by DDX58 or CXCL10 upregulation but not to exogenous dsDNA (Fig. 5e). In line with this, EtBr-treated cells demonstrated blunted CXCL10 expression after ER stress but not exogenous nucleic acid stimulation (Supplementary Fig. 4d).

MtDNA release is also a hallmark of apoptosis, both downstream of the UPR and due to other causes, and, at the same time, apoptotic cell death is also known to suppress mtDNA-mediated cGAS/STING activation^42,43^. Thus, we investigated whether caspase-3 activation correlated with type I IFN induction during ER stress. Using titrated amounts of all four ER stress activators we could observe that, while high levels of activation could efficiently induce apoptosis, these high levels were no efficient inducers of type I IFN release (as indicated by STAT1 phosphorylation) (Supplementary Fig. 4e). In contrast, lower levels of the compounds inducing ER stress could induce higher levels of STAT1 activation downstream of IFNAR but did not induce apoptotic cell death (Supplementary Fig. 4e). Thus, ER stress induces type I IFN release at subapoptotic levels which still allows for the release of mtDNA. Accordingly, despite ER stress, DM2 fibroblasts did not show signs of apoptosis, and the rate of cells in subG1 was below 0.5 percent (Supplementary Fig. 4f).

Since ATF6-deficiency blunted the type I IFN response downstream of ER stress, we then investigated whether direct activation of ATF6 also induced a type I IFN signature. To this end, we utilized both AA147, an antiviral compound described as activating ATF6^44,45^ (Supplementary Fig. 4g) as well as cells transduced with a lentiviral construct expressing ATF6(1-373)^46^ under a doxycycline-dependent promoter (Supplementary Fig. 4h). Both chemical activation of ATF6 with AA147 and ATF6N induction resulted in upregulation of CXCL10 in THP-1 cells. Moreover, while AA147-induced CXCL10 depended on expression of both cGAS and ATF6 in THP-1 cells (Supplementary Fig. 4g), ATF6N-induced CXCL10 could be inhibited by application of H151^47^, a small molecule inhibitor of cGAS/STING signaling (Supplementary Fig. 4i).

### Mitochondrial DNA release triggers cGAS dependent ISG induction in DM2

**Fig. 6:**
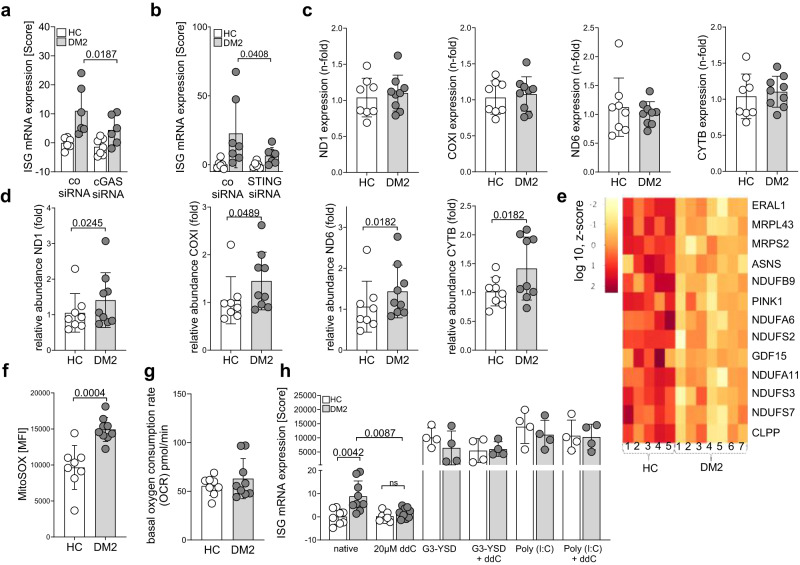
cGAS STING dependent ISG upregulation and mitochondrial stress in fibroblasts of DM2 patients. **a**, **b** IFN score^22^ was calculated from mRNA expression of the ISGs IFI44, IRF7, Viperin, Mx1, and DDX58 in fibroblasts of DM2 patients and healthy controls (HC) after siRNA knockdown of cGAS (**a**, DM2 *n* = 6, HC *n* = 7) and STING (**b**, DM2 *n* = 7, HC *n* = 6). **c** total mtDNA content in healthy controls (*n* = 8) and DM2 patients (*n* = 9) was determined by RT-PCR. **d** analysis of mtDNA content in cytosolic fraction of fibroblasts from healthy controls (*n* = 8) and DM2 patients (*n* = 9). **e** RNAseq analysis of DM2 (*n* = 7) and control (*n* = 5) fibroblast cell lines revealed significant downregulation of genes encoding mitochondrial respiratory chain complex I subunits (NDUFA6, A11, B9, S2, S3 and S7), proteins involved in mitochondrial gene expression (ERAL1, MRPL43, MRPLS2) and markers of mitochondrial stress responses (ASNS, PINK1, GDF15, CLPP). **f** fibroblasts of DM2 patients (*n* = 9) and controls (*n* = 8) were analyzed by flow cytometry using MitoSox. Mean fluorescence intensity (MFI) is shown. **g** measurement of basal oxygen consumption rate (OCR) in fibroblasts from DM2 patients (*n* = 9) and controls (*n* = 8). One representative experiment of three is shown. **h** fibroblast cells were treated with 20 µM ddC for 9 days to deplete mtDNA (HC: *n* = 8 DM2: *n* = 9). Cells were then stimulated with G3-YSD or Poly I:C (HC: *n* = 4 DM2: *n* = 4). ISG (IFI44, IRF7, Viperin, Mx1, DDX58) mRNA expression was determined by RT-PCR, and IFN score^21^ was calculated. **a**, **b** mRNA expression was determined using RT-PCR. Show data of two (**f**, **h**), three (**c**, **d**) or six (**a**, **b**) independent experiments. g, one representative experiment from three. Data are shown as mean ± SD. Statistical significance was assessed using student’s *t* test (**a**, **b**, **d**, **f**, **h**), paired student’s *t* test (**a**, **b**, **h**) or Mann-Whitney U test (**d**).

To analyze the impact of an ER-mitochondrial connection for ISG induction in DM2, we performed siRNA-mediated knockdown of cGAS and STING in DM2 fibroblasts (Supplementary Fig. 5a, b). Indeed, after downregulating cGAS or STING expression, the IFN score was significantly reduced in patient fibroblasts (Fig. 6a, b), indicating that the ISG signature in DM2 is cGAS and STING dependent.

The cytoplasmic DNA sensor cGAS can recognize self-DNA in the cytoplasm if this is released after mitochondrial outer membrane permeabilization (MOMP)^48^. To determine whether DM2 fibroblasts harbor mtDNA in their cytoplasm, we performed nuclear and cytoplasmic fractionation and assessed the mtDNA levels by PCR. While the total mtDNA content in patients and control fibroblasts was similar (Fig. 6c), the cytoplasmic fraction of patient fibroblasts contained elevated levels of mtDNA (Fig. 6d). Mitochondrial deterioration was further reflected by the reduction of mitochondrial membrane function associated genes in RNAseq (Fig. 6e, Supplementary Fig. 5c) and specific upregulation of mitochondrial ROS production (Fig. 6f). MOMP leading to DNA release is only compatible with life if it occurs in a limited number of mitochondria in the cell, during the so called minority MOMP event^49^. In line with this hypothesis, we did not detect general mitochondrial membrane depolarization using TMRM, a cell-permeant dye that accumulates in active mitochondria, nor a change in the total oxygen consumption rate in DM2 fibroblasts (Supplementary Fig. 5d–f, Fig. 6g). However, elimination of mtDNA by treating DM2 fibroblasts with 2’,3’ dideoxycytidine (ddC) (Supplementary Fig. 5g) ameliorated the ISG upregulation in patient fibroblasts but did not impair their capability to respond to innate danger signals like DNA (G3-YSD) or RNA (poly I:C) with ISG upregulation (Fig. 6h). These data indicate that the ISG upregulation in DM2 fibroblasts depends on mtDNA, in line with what was observed for THP-1 cells under ER stress (Fig. 5e). Furthermore, an increased presence of DNA in the cytoplasm was observed in skin sections of DM2 patients, which colocalized with cGAS (Supplementary Fig. 5h).

**Fig. 7:**
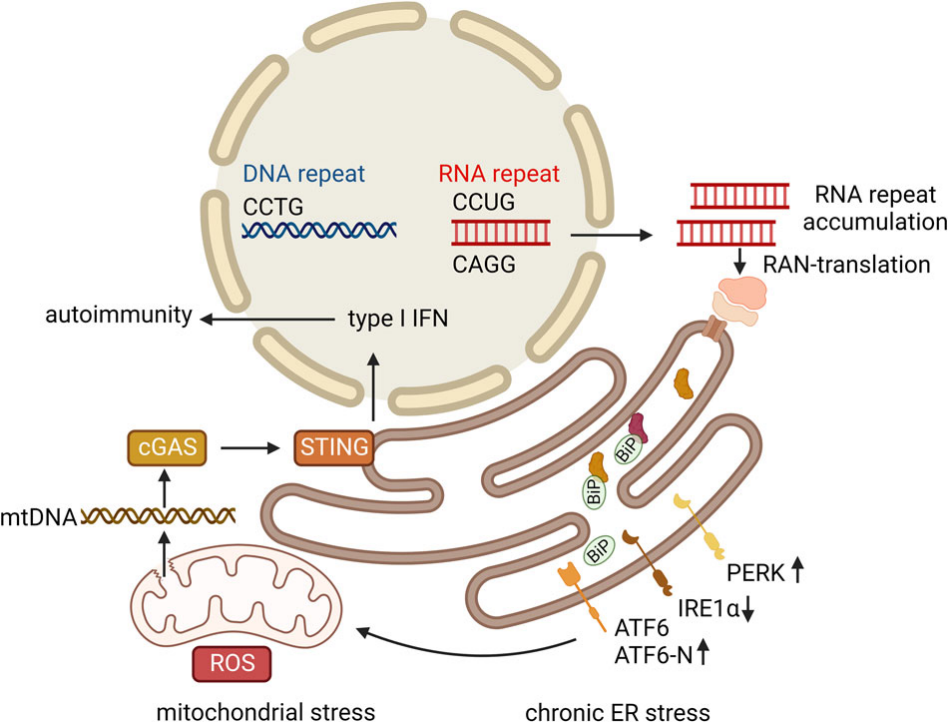
Graphical summary on the proposed mechanism inducing of autoimmunity in DM2. DM2 is characterized by CCTG repeat expansion in DNA that can be transcribed into RNA. RNA repeats accumulate in the nucleus and are transported into the cytoplasm. The cytosolic RNA repeats can be translated by repeat-associated non ATG (RAN) translation. These processes are associated with chronic ER stress indicated by increased BiP, PERK and ATF6-N expression. IRE1α is downregulated, which might be a consequence of ATF6 activation. Chronic ER stress leads to ATF6-dependent mitochondrial DNA release, ROS production and a cGAS-STING dependent upregulation of type I IFN and ISGs. Chronic type I IFN upregulation predisposes to autoimmunity in patients with DM2.

In conclusion we propose that chronic ER stress mediated by ATF6 activation allows cell survival at the expense of minority MOMP-mediated mtDNA release, leading to the activation of the cGAS/STING pathway and type I IFN-stimulated gene induction predisposing to autoimmunity (Fig. 7).

## Discussion

Here, we report that patients with myotonic dystrophy have an increased prevalence of concomitant autoimmunity associated with a cGAS/STING-dependent activation of the type I IFN response. First, we demonstrate that type I IFN-stimulated genes are upregulated in blood, lesional skin and isolated fibroblasts of patients with DM and especially DM2. This finding is further substantiated by the increased levels of autoantibodies in patients with DM2, which can be regarded as a first sign of autoimmunity. The prevalence of ANA in DM2 patients is elevated compared to healthy controls and is about twice as high as in the general German population (76% versus 36%, respectively)^50^. Three previous reports either demonstrated autoimmunity in DM2 patients^10,23^ or showed an ISG signature in cataract samples from DM2^51^ and thus support the association of DM2 with autoimmunity. The stable expression of ISGs in DM2 fibroblasts in culture in comparison with healthy controls indicate that a cell intrinsic mechanism is responsible for this type I IFN signature.

DM2 has the highest number of RNA repeats among all repeat expansion diseases. The number of repeat expansions ranges from 75 to over 11,000 repeats, which form stable RNA aggregates in the cell^1,9,52^. We demonstrated that these RNA repeats do not only accumulate in the nucleus but also can be frequently detected in the cytoplasm. This nuclear egress of repeat-expansion RNA has previously been reported in a model using CHO cells transfected with plasmids coding for a maximum of 100 CUG/CCUG repeats^12^ but not yet in patient cells that harbor much longer CCUG repeats. Unrestricted RNA can act as a danger signal in the cell, yet we did not observe evidence for direct sensing of repeat RNA by specific RNA sensors of the innate immune system. This could potentially be explained by self RNA modifications, such as mRNA cap methylation, and a lack of other specific RNA structures required for recognition by RIG-I, MDA5 or PKR^24^. However, we did observe that repeat RNA led to RAN translation and accumulation of nonsense proteins in fibroblasts and skin of patients, as previously described in the brain of DM2 patients^27^. These proteins can induce cellular stress, which may act as a positive feedback loop and further enhance RAN translation^27,53^. The upregulation of eIF2α and PERK indicates such a possible feedback loop because both factors have been recently shown to support RAN translation of LPAC and QAGR in DM2 as alternative initiation factors^54^. An active RAN translation might also be initiated by the ATP-dependent RNA helicase DHX36, which was upregulated at the mRNA level in patient cells and has been shown to facilitate RAN translation of C9orf72 GGGGCC repeat RNAs in cells of patients with amyotrophic lateral sclerosis (ALS)^55^. This hexanucleotide repeat expansion occurs in about 40% of familial cases of ALS and may drive disease pathogenesis due to either toxic gain of function or loss of normal function, formation of nuclear RNA foci that sequester a range of hnRNPs and production of poly-dipeptides (GA, GP, GR, PA, PR) through RAN translation^56^. Interestingly, these repeats have been shown to cause ER stress in a cellular overexpression model^28^. The response was characterized by activation of the PERK pathway. Differences in the UPR response between this ALS model and the UPR in DM2 might be explained by different cell types, repeat length, and potentially also differences in repeat sequence. In patient brains and iPSC-derived motoneurons of patients with mutation in C9orf72 ER stress was also detected indicating its relevance in disease pathogenesis^57,58^. Interestingly, this ER stress response was followed by reduced mitochondrial membrane potential and altered mitochondrial morphology^57^.

Repeat accumulation in fibroblasts from patients with DM2 was associated with a chronic ER stress response characterized by activation of the ATF6 pathway. The IRE1α pathway, which is typically induced by acute ER stress and leads to XBP1 splicing and IRE1α upregulation was not activated, and IRE1α itself was downregulated. Interestingly, it has been shown that ATF6 is involved in the downregulation of the protein IRE1α during sustained ER stress^37^. Upon ER stress, ATF6 traffics from the ER to the Golgi apparatus followed by a sequential cleavage^59^. The cleaved active form acts as a transcription factor of various genes controlling organelle homeostasis beyond ER stress^59^. Embryonic mouse fibroblasts deficient in the ER stress sensor ATF6 showed increased apoptosis and decreased adaptation to prolonged or recurrent stress^35^. Our finding in DM2 fibroblasts was substantiated by demonstrating that chronic stimulation of healthy fibroblasts by TG also induced selective ATF6 pathway activation, indicating that chronic and acute ER stress responses differ. Interestingly, chronic ER stress in THP-1 cells was associated with a similarly low-level type I IFN response as observed in DM2 fibroblasts and, most importantly, in both cell types, the ISG response was dependent on ATF6. Moreover, selective activation of the ATF6 pathway also resulted in low-level ISG induction. These findings demonstrate that chronic ER stress can lead to ATF6-dependent ISG upregulation in fibroblasts and myeloid cells.

The ER is closely connected with mitochondria and physical interactions between these organelles maintain mitochondria-ER contact sites^59^. The ER stress receptor PERK is part of the mitochondria-associated ER-membrane, and upregulation of this protein in DM2 fibroblasts might be relevant for stabilizing these connections^33,59^. Interestingly, we found that depletion of mtDNA completely abrogated the ISG response in THP-1 cells and fibroblasts, indicating that the ER-mitochondrial connection is required for ISG induction. It has been previously established that mitochondrial stress engages cytosolic antiviral signaling to enhance the expression of a subset of ISGs^60,61^. Aberrant mtDNA packaging promotes escape of mtDNA into the cytosol, where it engages the DNA sensor cGAS and promotes STING dependent ISG upregulation if not inhibited by apoptotic caspases^60^.

Importantly, elegant data by Ichim et al. have shown that mtDNA release is only compatible with survival if it is limited to a minority of mitochondria (minority MOMP)^49^ since the cell would otherwise die from caspase-initiated apoptosis. This concept is in line with our data, which demonstrate a relatively low amount of DNA in the cytoplasm and no change in the total mtDNA content. Moreover, we also observed a reduced expression of genes associated with mitochondrial membrane function, which corresponds to an independent proteomics analysis of myotubes derived from patients with DM2 which found that changes in protein expression belong to two major functional categories: mitochondrial components and ubiquitin proteasome system^62^.

The release of mtDNA has also been found to drive a growing number of other degenerative and autoinflammatory diseases via the cGAS/STING axis^63^ including Parkinson, amyotrophic lateral sclerosis and SLE^64–67^. However, these diseases or disease models are either driven by genetic defects in mitochondrial function and integrity or by the extrinsic uptake of DNA (e.g., after NETosis) into other cells. These studies undoubtedly present important mechanisms for mtDNA-mediated cGAS activation. Our work complements these findings, by demonstrating that chronic ER stress can induce a cell-intrinsic ISG response in the absence of additional mitochondrial defects and greatly expands the spectrum of potential diseases in which mtDNA sensing may act as substantial driver.

Altogether, we provide evidence for a new disease pathway that connects ATF6-controlled ER stress in DM2 fibroblasts with mtDNA release and ISG upregulation (Fig. 7). Elucidating this pathway opens new avenues for understanding other illnesses, both monogenetic and multifactorial, that are accompanied by increased ER stress. These include other nucleotide expansion diseases, such as polyQ diseases^68^, as well as metabolic disturbances such as type 2 diabetes mellitus and inflammation during obesity^69–71^. Of note, the proinflammatory western diet has been associated with both ER stress and ISG upregulation although these phenomena have not been linked mechanistically to date^72,73^. Further studies will be needed to elucidate the role of ATF6-dependent type-I IFN induction in ER stress-mediated pathogen defense as well as human disease.

Chronic, low-level activation of the ISG response in cells of DM2 patients likely promotes the manifestation of autoimmune diseases. We know that autoimmunity can be induced by chronic low-level ISG induction in monogenic interferonopathies caused by mutations that impair intracellular restriction of nucleic acids^16^. For example, familial chilblain lupus is caused by loss-of-function mutations in the DNase TREX1^74,75^ which induce accumulation of DNA in the cytoplasm that is sensed by the cGAS-STING-pathway and leads to chronic low level ISG upregulation^76^. Trigger factors such as UV-irradiation or cold exposure enhance the type I IFN response and elicit diseases flares^77^. Similarly, SLE is promoted by mutations in RNASEH2 that impair ribonucleotide excision from DNA and cause DNA damage and repair-associated chronic ISG upregulation^20^. Thus, our findings place DM2 among other autoimmune diseases resulting from cGAS/STING-induced chronic ISG upregulation and autoimmunity and into the larger context of type I interferon-driven disease.

Importantly, our data also demonstrate that, like other type-I IFN-associated diseases, DM2-associated autoimmunity is potentially druggable by compounds interfering with type I IFN activation such as Janus kinase inhibitors or IFNAR receptor blockers, opening new avenues for the treatment of this disease.

## Methods

### Study approval

Patients with DM2 and DM1 and healthy controls were enrolled after written, informed consent. Human primary fibroblasts were derived from skin biopsies. Control samples were obtained from skin discarded during plastic surgery. The study was approved by the ethics committee of the Medical Faculty, Technische Universität Dresden.

### Cell culture and stimulation

Fibroblasts of 9 DM2 patients and 8 healthy controls were cultured in DMEM (Gibco) supplemented with 10% FCS, 1% antibiotics and 1 mM sodium pyruvate. In all experiments passage-matched cells (passages 6−13) were used. For stimulation of fibroblasts with poly I:C, 10 µg/ml (Invivogen #tlrl-pic) was used. Poly I:C was diluted in medium and incubated for 3 h. To induce chronic ER stress, fibroblasts were seeded in 6-well plates and incubated with 5 nM, 1 nM or 0.2 nM thapsigargin (Cayman Chemical Company) for seven days. The medium containing thapsigargin was changed every two days. Acute ER stress was induced in fibroblasts by incubation with 50 nM, 25 nM, 5 nM, 1 nM or 0.2 nM thapsigargin for 6 h. For stimulation with cGAS agonist G3-YSD^78^ (InvivoGen #tlrl-ydna), 0.2 mg/ml was transfected by Lipofectamine 2000 according to the manufacturer’s instructions, and then cells were incubated for 24 h.

THP-1 cells were cultivated in RPMI supplemented with 10% FCS, 1% antibiotics and 1 mM sodium pyruvate. dsDNA(1 µg/mL) and 3pRNA(200 ng/mL) were complexed with Lipofectamine 2000 (Invitrogen) prior to transfection according to the manufacturer’s instructions. The ER stress inducers cyclopiazonic acid (Cayman Chemical Company), 2,5-di-*t*-butyl-1,4-benzohydroquinone (Merck/Sigma-Aldrich), thapsigargin (Cayman Chemical Company) and tunicamycin (Merck/Sigma-Aldrich) were added directly to the cell culture medium at the concentrations indicated in the respective subfigures. Supernatants were harvested for ELISA or RT-PCR 24 h after stimulation.

Wildtype, STING^-/-^ and cGAS^-/-^ HT29 cells were provided by Katarzyna Andryka and cultivated in Dulbecco’s modified Eagle medium (DMEM) with 10% fetal calf serum (FCS), 1% non-essential amino acids (NEAA), 1% sodium pyruvate, 100 IU/ml penicillin and 100 µg/ml streptomycin. Stimulation of the cells was performed as for THP-1 in the methods section: dsDNA(1 μg/mL) and 3pRNA(200 ng/mL) were complexed with Lipofectamine 2000 (Invitrogen) prior to transfection according to the manufacturer’s instructions. The ER stress thapsigargin (Cayman Chemical Company) and tunicamycin (Merck/Sigma-Aldrich) were added directly to the cell culture medium at the concentrations indicated. Supernatants were harvested for ELISA 24 h after stimulation.

### Autoantibody testing

Routine serological tests were carried out at the diagnostic laboratory of the Department of Dermatology and Institute of Immunology, Technische Universität Dresden. ANAs were determined using Hep-2 cells; extractable nuclear antigens were analyzed by immunoblot. Data on ANAs from a reference population were obtained from 1000 blood donors (samples collected at the Institute of Immunology, Technische Universität Dresden)^20^.

### RT-PCR

Total RNA or DNA from fibroblasts was extracted with the RNeasy Mini Kit (Qiagen) followed by DNase I digestion or DNeasy blood & tissue kit (Qiagen 69504 and 69506). Total RNA from blood was extracted with the PAXgene Blood RNA Kit (PreAnalytiX #762174). mRNA expression of IFNβ, DHX36, CNBP, cGAS, STING, MAVS, MDA5,ISGs (IFIT1, IFI44, IFI44L, CXCL10, ISG15, IFI27, Viperin, IFI16, IRF7, TLR3, Mx1, DDX58) and ER stress factors (BiP, ATF6, XBP1 spliced, XBP1 unspliced, PERK) were determined using iQ SYBR Green Supermix (Bio-Rad #1725124) on an Mx3005P RT-PCR system (Agilent) and normalized to HPRT1 (Supplementary Table 5). The IFN score was calculated as described by ref. ^22^: $\sum=\frac{({Gene}\,{of}\,{interest}-{Gene}\,{of}\,{control})}{{SD}({Gene}\,{of}\,{control})}$

### Immunohistochemistry

Paraffin-embedded skin biopsies were cut into 2 to 5-μm sections, rehydrated, and boiled in sodium citrate buffer (pH 6.0). Sections were stained with mouse anti-MxA (provided by O. Haller, Freiburg University, Breisgau, Germany; 1:400 dilution) followed by staining with EnVision G | 2 System/AP Rabbit/Mouse (Dako) or antibodies against LPAC (Merck ABN2258, 1:15000) and QAGR (Merck ABN2271, 1:20000). Sections were counterstained with Mayer’s hematoxylin (Merck).

### Cytokine detection

IFNβ secreted to the supernatants of fibroblasts was quantified using the HEK-Blue^TM^ IFN-α/β reporter system by InvivoGen and normalized to the cell number. Cell number was determined by Hoechst 33258 staining. CXCL10 release was measured using the human IP10 ELISA set (BD Bioscience), performed according to the manufacturer’s instructions.

### RNA-FISH

Cells were seeded in a 24-well plate. Fibroblasts were fixed with 3.7% formaldehyde. Permeabilization was performed using 70% ethanol. RNase A (Thermo Scientific, #EN0531) treatment followed for 1 h at room temperature. Hybridization of the (CAGG)8 (Eurofins) probe was then performed at 37 °C overnight. Cells were mounted in antifade medium containing DAPI (Thermo Scientific). Cells were imaged using Perkin Elmer Operetta System. The imaging settings were 4 planes per position (DAPI, GFP, mCherry), 40 × 0.95 NA objective. Images were then analyzed using the software Cellprofiler (version 3.1.8).

### siRNA transfection

Fibroblasts were transfected with 10 nM of RIG-I (Invitrogen 10620319-360113), MDA5 (Invitrogen 10620319-348588), MAVS (Invitrogen 10620319-361473), TLR3 (Invitrogen 10620319-367493), STING (Invitrogen 10620319-361473), cGAS (Invitrogen 10620319-383441), PERK (Invitrogen 21255167) or ATF6 (Invitrogen 10620319-439921) siRNAs. According to the guanine content of the individual siRNAs, the cells were transfected with medium or high control siRNAs (Invitrogen) using Lipofectamine®2000 or Lipofectamine® RNAiMAX (Invitrogen). Cells were prepared 72 h after transfection for RT-PCR.

### Immunoblotting

Fibroblasts were lysed in 2x Laemmli buffer (125 mM Tris/HCl, pH6.8, 4% SDS, 10% glycerol, 0.02% Bromophenol blue) or RIPA buffer (50 mM Tris-HCl, pH 7.4, 150 mM NaCl, 1 mM EDTA, 1% Triton X-100, 1 mM sodium orthovanadate, 20 mM sodium fluoride) supplemented with 1× Complete Protease Inhibitor Cocktail (Roche) and 1× PhosSTOP phosphatase inhibitors (Roche). THP-1 cells were lysed using 1x Laemmli buffer. 20 μg total protein was subjected to SDS-PAGE electrophoresis followed by Immunoblotting using antibodies against LPAC (Merck ABN2258, 1:1000), PERK (Cell Signaling #5683, 1:500) ATF6 (Cell Signaling #65880, 1:500), ATF6-N (Novus biologicals 75478, 1:500), IRE1α (Cell Signaling #3294, 1:500), eIF2α (Cell Signaling #9722, 1:1000), peIF2 α (Cell signaling #3398, 1:1000) and GAPDH (Cell Signaling #2118, 1:1000), β-actin (Cell Signaling #4970, 1:1000, Santa Cruz sc-47778, 1:2000 and Licor 926-42212, 1:1000), α-Tubulin (Neomarker MS-581-P1, 1:1000) PKR (Cell Signaling #12297, 1:1000), phospho-STAT1 (Cell Signaling #9167, 1:1000) and cleaved caspase 3 (Cell Signaling #9661, 1:1000), HA-tag (ThermoFisher Scientific PA1-29751, 1:500), pPKR (Abcam ab32036, 1:1000), CNBP (Sigma SAB2100453, 1:200) and DHX36 (santa cruz sc-377485, 1:500).The antibodies IRDye 680RD goat anti- mouse IgG (Licor 926-68070, 1:5000), Anti-rabbit IgG Peroxidase antibody produced in goat (Sigma A0545, 1:3000) and ECLTM Anti-mouse IgG, Horseradsh Peroxidase linked whole antibody (from sheep) (Cytiva NA931V, 1:3000) were used as secondary antibodies. Immunoreactive signals were detected by chemiluminescence (Super Signal West or Super Signal Pico; Thermo Scientific). Images were taken on Image Quant LAS 4000 (GE Healthcare).

### Proliferation

Seeding was done simultaneously for four different time points (day 0, 3, 5, 7). The cells were incubated at 37 °C until the specific time point and then fixed with 4% formaldehyde for 10 min, followed by a treatment with 0.25% TritonX-100 for 10 min, both at room temperature. Fibroblasts were then treated with Hoechst 33258 (5 µg/ml) for 15 min at room temperature before measurement on a microplate fluorometer. The cell number for each well was determined based on a standard curve using set numbers of cells.

### ROS detection

For detection of ROS cells were incubated with dihydrorhodamine 123 (DHR 123, Molecular Probes, 1 µg/ml, ChemCruz sc-203027) in DMEM without phenol red. After incubation for 15 min at 37 °C, ROS-induced fluorescence was measured on a Tecan microplate reader (excitation 488 nm, emission 530 nm).

### Analysis of RNA-Sequencing data

Within the framework of the bioinformatic workflow, raw reads were inspected using fastqc (https://www.bioinformatics.babraham.ac.uk/projects/fastqc/), trimmed using trimmomatic (https://academic.oup.com/bioinformatics/article/30/15/2114/2390096?login=true) and aligned using STAR (https://academic.oup.com/bioinformatics/article/29/1/15/272537?login=true), GRCh37 was used as reference genome. Read counts were extracted from the alignments using the featureCounts method of the subread package (https://academic.oup.com/bioinformatics/article/30/7/923/232889). Afterwards, DESeq2 was applied to identify differentially expressed genes (https://genomebiology.biomedcentral.com/articles/10.1186/s13059-014-0550-8). Only genes with multiple testing adjusted *p* (padj from DESeq2) < 0.05 were considered significant. The Interferome database was used to identify ISGs^79^. Heatmaps were created using RStudio with the plugin heatmap.2.

### β-galactosidase staining

Fibroblasts were synchronized by serum starvation for 24 h. The detection of senescence was performed with the Senescence Detection Kit from BioVision (Biozol #K320-250). To enable long-term storage at 4 °C, 1 ml of 70% glycerol was added to the cells. Plates were analyzed under a light microscope. For each well, four areas were defined, and the blue stained and non-stained cells were counted manually.

### CRISPR/Cas9

ATF6A, ATF6B, cGAS, EIF2AK3, ERN1, IRF3, MAVS and STING gRNAs (Supplementary Table 4) were selected with the CRISPR design tool (Zhang Lab, MIT, crispr.mit.edu) and introduced into an EF1a-Cas9-U6-sgRNA expression plasmid via Gibson assembly. Single-cell clones were obtained by limiting dilution plating; loss of expression was confirmed by immunoblotting, and InDels were determined by Sanger sequencing (Supplementary Table 4). The cGAS and MAVS deficient THP-1 clones were generated as described above and have been published previously^80^.

### mtDNA-depletion

THP-1 cells: Mitochondrial DNA (mtDNA) was depleted by incubation of wild type (WT) THP-1 cells with 5 µM of IMT1^40^ in cell culture medium supplemented with 200 µM uridine and 2 mM sodium pyruvate. After 14 days, mtDNA depletion was assessed using RT-PCR for MT-ND1, MT-ND5 and MT-ND6, with ACTB as a reference gene for nuclear DNA (Supplementary Table 5). In addition, ethidium bromide (EtBr) was used to deplete mtDNA in THP-1 cells as described in^41^. In brief, THP-1 cells were incubated for eight weeks in 50 ng/ml EtBr, and mtDNA depletion was assessed using RT-PCR for B2M and MT-ND1, which were used as reference genes for nuclear DNA and mtDNA, respectively.

Fibroblasts: fibroblasts were incubated with 20 µM 2’,3’ dideoxycytidine (ddC) for 9 days to deplete mtDNA. After 9 days, RT-PCR for CYTB, COXI and ND6 was performed to verify the depletion of mtDNA, with ACTB as a reference gene for nuclear (Supplementary Table 5).

### Subcellular fractionation

5 × 10^5^ cells were seeded in 10 cm petri dishes and incubated at 37 °C for three days. After incubation, fibroblasts were detached using trypsin EDTA, washed with PBS, and incubated on a rotator for 10 min at 4 °C in fractionation buffer (150 M NaCl, 15 mM HEPES pH 7.4,1x Protease Inhibitor, 20 µg/ml digitonin, DEPC water).The mixture was then centrifuged at 1000 x g for 3 min, and the supernatant was decanted and centrifuged at 17,000 x g for 10 min. This step was repeated once. The supernatant after the last centrifugation represents the cytoplasmic fraction. DNA was isolated from the cytosolic fraction using the Qiagen DNeasy blood & tissue kit (cat. no. 69504 and 69506). Nuclear fraction was used as a control. Expression of mtDNA genes (ND1, COXI ND6, CYTB, ND5) was determined by RT-PCR and normalized to ACTB (Supplementary Table 5).

### FACS staining

To detect mitochondrial stress, 10^5^ fibroblasts were incubated for 30 min at 37 °C with MitoSOX^TM^ (5 µM, Invitrogen M36008) or Image-iT^TM^ TMRM Reagent (100 nM, Invitrogen 134361). After incubation, the staining was analyzed by flow cytometry on a FACS Canto II instrument. The analysis of the data was performed using FlowJo software (Supplementary Fig 6).

For the propidium iodide staining fibroblasts were seeded at a density of 10^5^ cells per well in a 6 well plate. For DNA content analysis, cells were collected after 24 h, fixed/permeabilized with ice cold 75% ethanol for 1 h at 4 °C and stained with propidium iodide/RNase solution (50 µg/ml propidium iodide and 100 µg/ml RNase in PBS). Flow cytometry analysis was performed on a LSR Fortessa (Becton Dickenson, Franklin Lakes).

### Seahorse assay

Oxygen consumption rate (OCR) was measured in fibroblasts with Seahorse XFe96 Analyzer (Agilent Technologies) using Seahorse XF Cell Mito Stress Test Kit (103015-100). Fibroblasts (1.5 × 10^4^) were seeded in Agilent Seahorse XF96 cell culture microplates. The assay was carried out in assay medium containing 1mN pyruvate, 2 mM glutamine and 10 mM glucose. The modulators used were Oligomycin (2.5 µM), Carbonyl cyanide-4 (trifluoromethoxy) phenylhydrazone (FCCP, 1 µM), Rotenone (0.5 µM) and Antimycin (0.5 µM). The cell count was normalized to the average value. To achieve this, cells were stained after the assay using the DNA marker DAPI and images were taken on the Keyence BZ-X800 microscope. Cell numbers were counted by hand and normalized using Wave software.

### G-quadruplex staining

Fibroblasts were fixed using methanol and acid (3:1) for 10 min at room temperature. This was followed by permeabilization of the membrane using PBS containing 0.1% Triton X-100, by treatment of the fibroblasts with 2% skim milk for 1 h at room temperature, and by incubation of the fibroblasts with the G-quadruplex antibody with a FLAG tag (BG4 2 µg) for 2 h. The BG4 antibody was produced by Katrin Paeschke, Bonn, Germany and kindly provided to us. Incubation with anti-flag antibody for one hour (Cell Signaling 2368 S, 1:800) was followed by incubation with the appropriate secondary antibody (goat a-rabbit IgG Alexa Fluor 488 (LifeTechnologies, #11008 A, 1:1000)). 50 cells of each cell line were analyzed at Axio Imager A1 from Zeiss.

### ELISA

RNA from DM2 patients was transfected into MDA5^-/-^ Hela cells. After 24 h the supernatant was harvested, and the concentration of CXCL10 was determined by ELISA (ELISA MAX™ Deluxe Set Human CXCL10 (IP-10) #439904 BioLegend, San Diego, CA). For THP-1 experiments, the human IP10 ELISA set (BD Bioscience) was used.

### THP1-Dual^TM^ cells

RNA from 5 DM2 patients and 5 healthy controls was transfected into THP1-Dual^TM^ cells (InvivoGen). Poly I:C and 5’ triphosphorylated RNA (3pRNA) were used as positive controls. To determine the IFN induction, the activity of luciferase was measured using QUANTI-Luc^TM^ (InvivoGen).

### ATF6 activation studies

THP-1 cells were incubated for 24 h with the ATF6 activator AA147 (CAS 393121-74-9, Tocris Bioscience, Bristol, UK) prior to RT-PCR. For lentiviral transduction of ATF6N, cells were transduced using a modified pLIX with a tetracycline-inducible promoter provided by Prof. Andreas Pichlmair (TU Munich), containing a HA-tagged ATF6N construct subcloned from pCGN-ATF6 (1-373) (Addgene #27173) or a control construct with mutated ATF6 DNA binding domains pCGN-ATF6 (1-373)m1 (Addgene #27174). Both pCGN plasmids were a gift from Ron Prywes. Lentivirus was generated as previously described^38,81^. In brief, HEK293T cells were transfected with a lentiviral vector, a plasmid expressing VSV-G (pMD2.G, Addgene #12259) and a lentiviral packing plasmid (pspax2, Addgene #12260) using HBSS/CaCl2 (calcium phosphate method). Both pMD2.G and pspax2 were a gift from Didier Trono and the Trono lab. Viral supernatants were concentrated using ultracentrifugation and then used to spin transduce THP-1 cells (32 °C, 900 g, 60 min). One week after transduction, THP-1 cells that had integrated the lentiviral expression cassette were positively selected using puromycin. Expression of the ATF6 constructs was tested by exposing transduced THP-1 cells to titrated levels of doxycycline, followed by immunoblotting of cell lysates using an anti-HA antibody (PA1-29751, ThermoFisher Scientific, 1:500).

### Immunofluorescence staining

Paraffin-embedded skin biopsies were cut into 2 to 5 μm sections, rehydrated, and boiled in sodium citrate buffer (pH 6.0). Followed by permeabilization of the membrane using PBS containing 0.2% Triton X-100. After treatment of the cells with blocking buffer (3% BSA in 1x PBS), the sections were incubated with the primary antibody mouse anti-DNA (Progen #61014, 1:40) and anti-cGAS Novus Biologicals (#NBP1-86767, 1:200) overnight at 4 °C, followed by incubation with the appropriate secondary antibody goat anti-mouse IgM AF488 (LifeTechnologies, #A-21042, 1:200); goat anti-rabbit IgG-AF546 (LifeTechnologies, #A-11071, 1:200) for 30 min at room temperature. The images of the skin sections were taken with the Axio Imager A1 from Zeiss.

### Statistical analysis

Data are presented as mean ± SD and representative of at least three independent experiments unless otherwise was indicated. Statistical analysis was performed using Graphpad prism version 9.3.1. The normality of distributions was tested using the Shapiro Wilk test. In normally distributed samples, the unpaired two-tailed student’s *t* test was used for comparison of two groups. Samples that were not normally distributed were analyzed by Mann Whitney U test for comparison of two different groups. The comparison of normally distributed equal groups was calculated by paired two-tailed student’s *t* test. For non-normally distributed samples, the Wilcoxon test was used. *p* < 0.05 were considered statistically significant. For multiple comparisons, the two-way ANOVA was used for normal distributed samples or the Kruskal-Wallis for a non-normal distribution. The Bonferroni post hoc test was applied for the two-way ANOVA, the Dunn’s post hoc test was applied for Kruskal-Wallis and the Holm Sidak post hoc test was used for multiple-paired *t* tests. Images were created with full licensed BioRender.com.

### Reporting summary

Further information on research design is available in the Nature Portfolio Reporting Summary linked to this article.

### Supplementary information

Supplementary Information

Reporting Summary

Description of Additional Supplementary Files

Supplementary Data 1

### Source data

Source Data

## Data Availability

The RNA-Sequencing data have been deposited in Gene Expression Omnibus (GEO) database under accession code GSE242388. Source data are provided with this paper.
