# Supplementary material for: Chronic endoplasmic reticulum stress in myotonic dystrophy type 2 promotes autoimmunity via mitochondrial DNA release
Source: Nat Commun. 2024 Feb 20;15:1534. doi: 10.1038/s41467-024-45535-1 (PMC10879130; doi:10.1038/s41467-024-45535-1)
Supplement: Supplementary file 1 — Supplementary Information [file 41467_2024_45535_MOESM1_ESM.pdf]

Chronic endoplasmic reticulum stress in myotonic dystrophy type 2 promotes autoimmunity via mitochondrial DNA release

Rösing Sarah[1], Ullrich Fabian[2,3], Meisterfeld Susann[1], Schmidt Franziska[1], Mlitzko Laura[1], Croon Marijana[4], Nattrass Ryan George[3], Eberl Nadia[1], Mahlberg Julia[2], Schlee Martin[2], Wieland Anja[2], Simon Philipp[2], Hilbig Daniel[5], Reuner Ulrike[6], Rapp Alexander[7], Bremser Julia[3], Mirtschink Peter[8], Drukewitz Stephan[9], Zillinger Thomas[2], Beissert Stefan[1], Paeschke Katrin[5,2], Hartmann Gunther[2], Trifunovic Aleksandra[4], Bartok Eva[2,3,10,*], Günther Claudia[1,*,#]

[1]Department of Dermatology, University Hospital Carl Gustav Carus, TU Dresden, 01307 Dresden, Germany

[2]Institute of Clinical Chemistry and Clinical Pharmacology, University Hospital Bonn, 53127 Bonn, Germany

[3]Institute of Experimental Haematology and Transfusion Medicine, University Hospital Bonn, 53127 Bonn, Germany

[4]Institute for Mitochondrial Diseases and Aging, Faculty of Medicine, CECAD Research Center, 50931 Cologne

[5]Department of Oncology, Hematology, Rheumatology and Immune-Oncology, University Hospital Bonn, 53127, Bonn, Germany

[6]Department of Neurology, University Hospital Carl Gustav Carus, TU Dresden, 01307 Dresden, Germany

[7]Department of Biology, Cell biology and Epigenetic, Technical University of Darmstadt, Darmstadt, Germany

[8]Institute for Clinical Chemistry and Laboratory Medicine, Faculty of Medicine, TU Dresden, 01307 Dresden, Germany

[9]Core Unit for Molecular Tumor Diagnostics (CMTD), National Center for Tumor Diseases (NCT), Partner Site Dresden, Institute of Human Genetics, University of Leipzig Medical Center, Leipzig, Germany

[10]Unit of Experimental Immunology, Department of Biomedical Sciences, Institute of Tropical Medicine, Antwerp, Belgium

*equal contribution

[#]Address correspondence to: Claudia Günther, Department of Dermatology, Medizinische Fakultät Carl Gustav Carus, Technische Universität Dresden, Fetscherstr. 74, 01307 Dresden, Germany. Phone: 0049.351.458 2344; E-mail: claudia.guenther@ukdd.de

Supplementary Figures

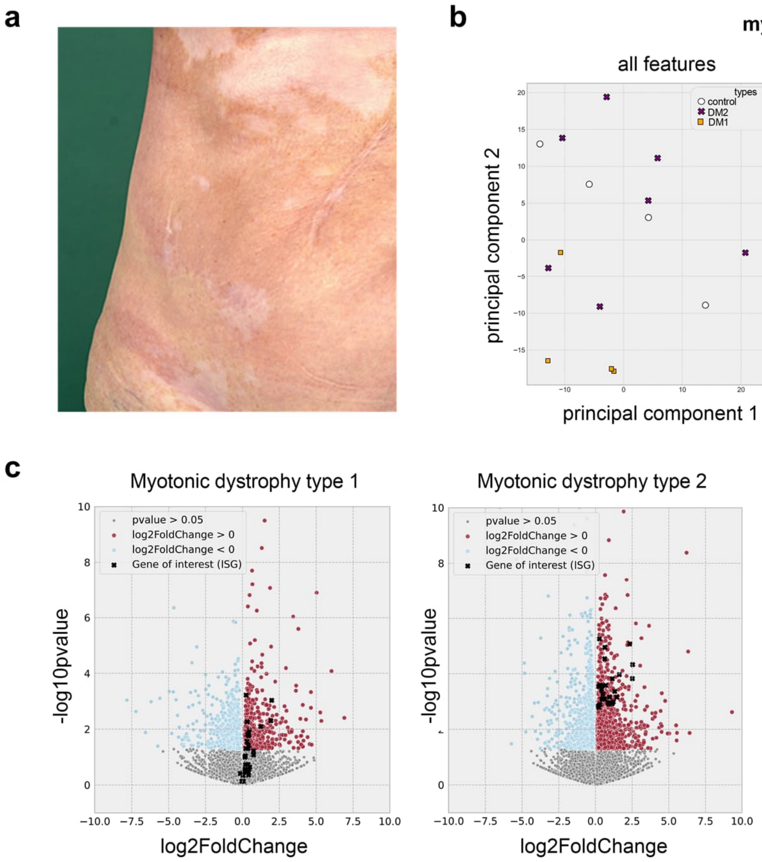

Supplementary Figure 1: Activation of a type I IFN response and autoimmunity in patients with myotonic dystrophy. a, Patient developed erythematous patches that progressed to firm plaques with ivory centers covering mainly the trunk. Histology was consistent with morphea. Patient additionally developed vitiligo. **b**, Principal component analysis of 7 DM2, 4 DM1 patients and 5 healthy controls. **c,** Volcano Blots showing all significantly differentially regulated genes in 4 DM1 (left) and 7 DM2 (right) patients compared to 5 healthy controls. ISGs are marked with a black cross.

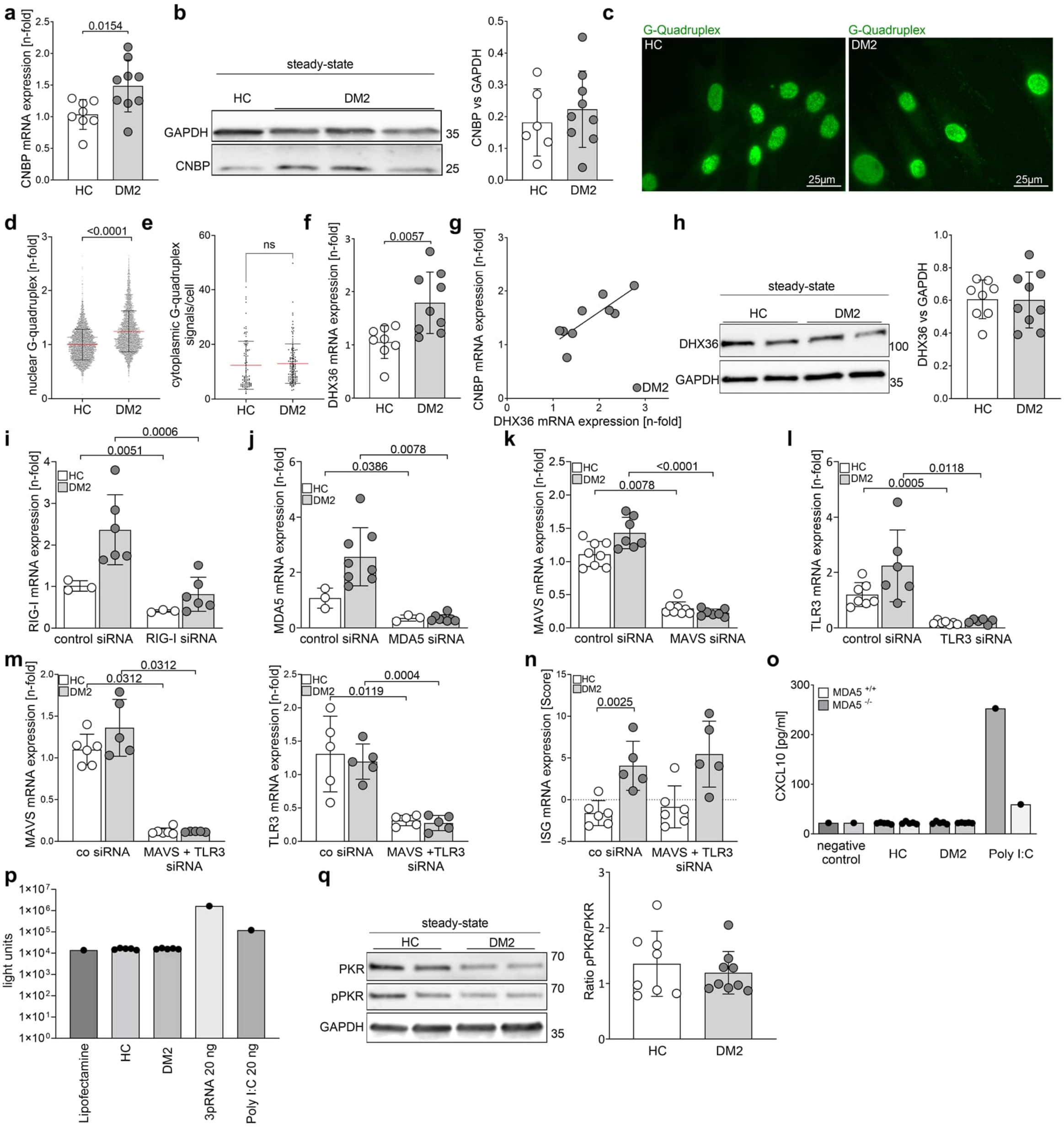

Supplementary Figure 2: Control experiments for G-quadruplex (G4) structures and RNA immune sensing. **a**, relative mRNA expression of CNBP in HC (n=8) and DM2 patients (n=9). **b**, immunoblots of CNBP (left) and quantification of all samples HC (n=6) and DM2 patients (n=9). **c**, images of immunofluorescence staining of G4 structures. **d**, **e**, quantification of nuclear and cytoplasmic G4 structures in HC (n=6) and DM2 (n=9) patients. **f**, relative mRNA expression of DHX36 in HC (n=8) and DM2 patients (n=9). **g**, correlation between the mRNA expression of CNBP and DHX36 genes. **h**, immunoblots of DHX36 and quantification of samples (n=8 HC and n=9 DM2 patients). **i-m**, mRNA expression of RIG-I (**i**), MDA5 (**j**), MAVS (**k**) and TLR3 (**l**) after siRNA-mediated single knockdown or double knockdown of MAVS and TLR3 (**m**). **n**, calculated IFN score[1] after siRNA double knockdown of MAVS and TLR3. **o**, **p,** RNA isolated from fibroblasts of DM2 patients (n=5) containing RNA-repeats and HC (n=5) was transfected into (**o**) MDA5 expressing and MDA5 non-expressing Hela cells or (**p**) THP1 dual sensor cells. Poly I:C was used as a positive control for MDA5 activation, and 5' triphosphorylated RNA (3pRNA) as positive control for RIG-I. (**o**) The CXCL10 concentration in the supernatant was measured by ELISA. One representative experiment out of three is shown. (**p**) Luciferase activity was determined by Quanti-Luc to quantify the activation of the

IRF3 pathway. One representative experiment of three is shown. **q**, immunoblot analysis of protein kinase R (PKR) and phosphorylated PKR (pPKR) protein levels in fibroblasts of DM2 patients (n=9) or healthy controls (n=8). The ratio of pPKR and PKR is indicated. a, f, i-m, mRNA expression was determined using RT-PCR. a-n, q, include data from one (b, d, e) two (m, n), three (f, i, j, g) four (a, h, k, l) independent experiments. o and p, representative of five DM2 patients. Data are shown as mean ± SD. Statistical significance was assessed using student's t-test (a, f, n), paired student's t-test (i, j, k, l, m, n), Wilcoxon (i, j, k, m), one-tailed Wilcoxon (m) or Mann-Whitney U test (d).

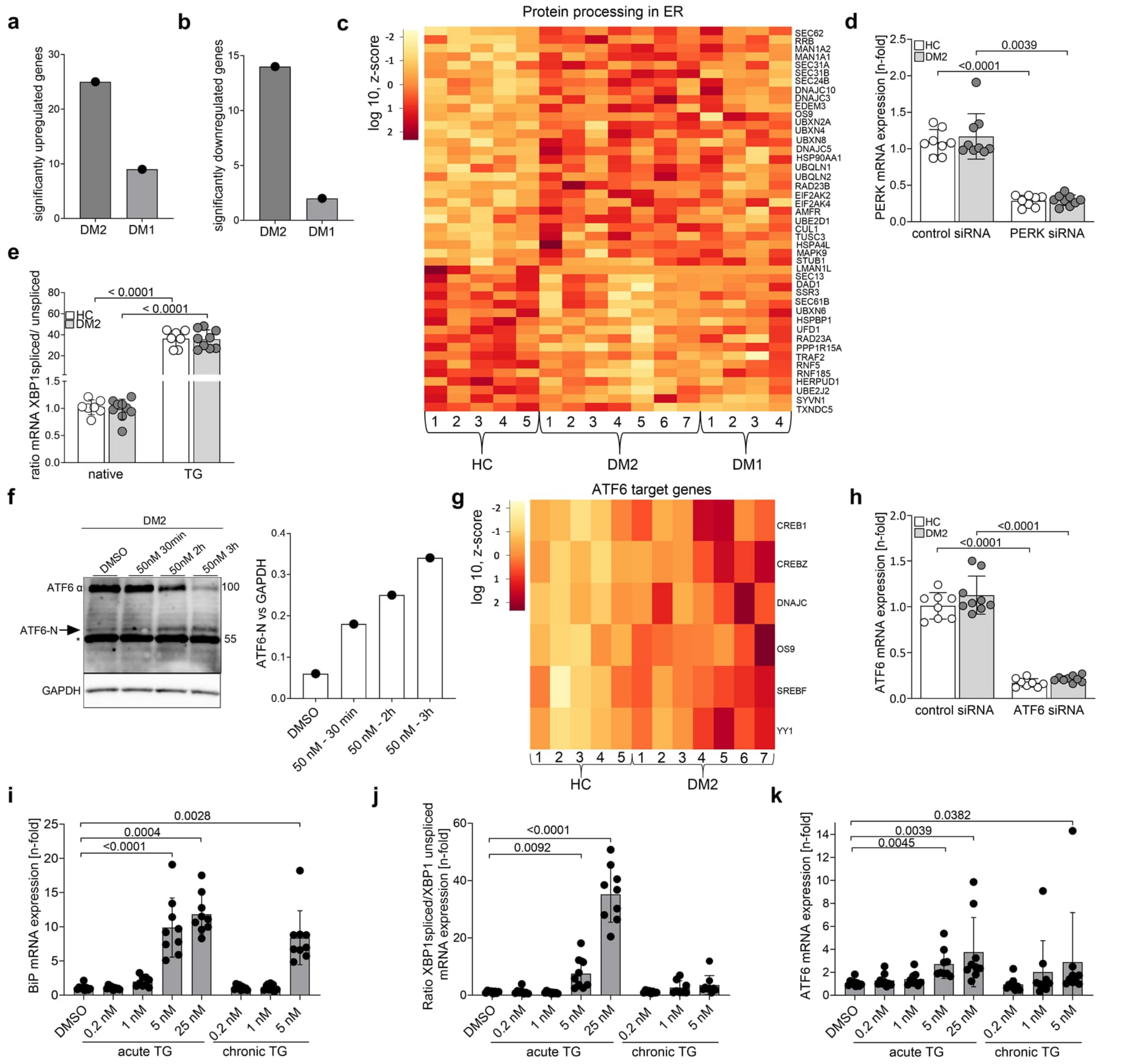

Supplementary Figure 3: Analysis of ER function in myotonic dystrophy type 2 patients. a, **b**, total number of significantly upregulated (**a**) and downregulated (**b**) genes involved in ER stress in DM1 and DM2 patient fibroblasts, based on RNAseq with n=4 DM1, n=7 DM2 and n=5 control fibroblast cell lines and analyzed using the KEGG pathway “Protein processing in ER”. **C**, heatmap of the differential expression of the genes from the RNAseq in (a, b). Genes displayed in this heatmap are also shown in the heatmap of Figure 4d. **d**, mRNA expression of PERK after siRNA knockdown in n=8 HC and n=9 DM2 patients. **e**, ratio of unspliced and spliced XBP1 relative mRNA expression under native conditions and after TG (50nM) stimulation in n=8 controls and n=9 DM2 patients. **f**, fibroblasts were stimulated with 50nM TG for 30 min, 1h or 3h. Activation of the ATF6 pathway is indicated by cleavage of ATF6 and increased detection of the ATF6-N band, *denotes unspecific bands. The right figure demonstrates the quantification of ATF6N in the representative immunoblot on the left. **g**, significantly upregulated ATF6 target genes determined by RNAseq analysis in fibroblasts of n=5 healthy controls and n=7 patients with DM2 are shown. **h**, mRNA expression of ATF6 after siRNA knockdown in n=8 HC and n=9 DM2 patients. **i-k**, relative mRNA expression of BiP (**i**) XBP1 (**j**) and ATF6 (**k**) in fibroblasts. To induce acute ER stress, fibroblasts were treated once with 25 nM, 1nM, 5nM and 0.2nM TG. For chronic ER stress, fibroblasts were treated with 5nM, 1nM or 0.2nM TG for one week. D, E, H, I-K, mRNA expression was determined using RT-PCR. d-f, h, include data from one (f) or three (d, h, e) independent experiments. i-k,

representative of 4 healthy donors. Data are shown as mean ± SD. Statistical significance was assessed using student's t-test (h), paired student's t-test (d, e, h), Kruskal Wallis and the Dunn's post-hoc test (i,j) or one-tailed Kruskal Wallis and the uncorrected Dunn's post hoc test (k).

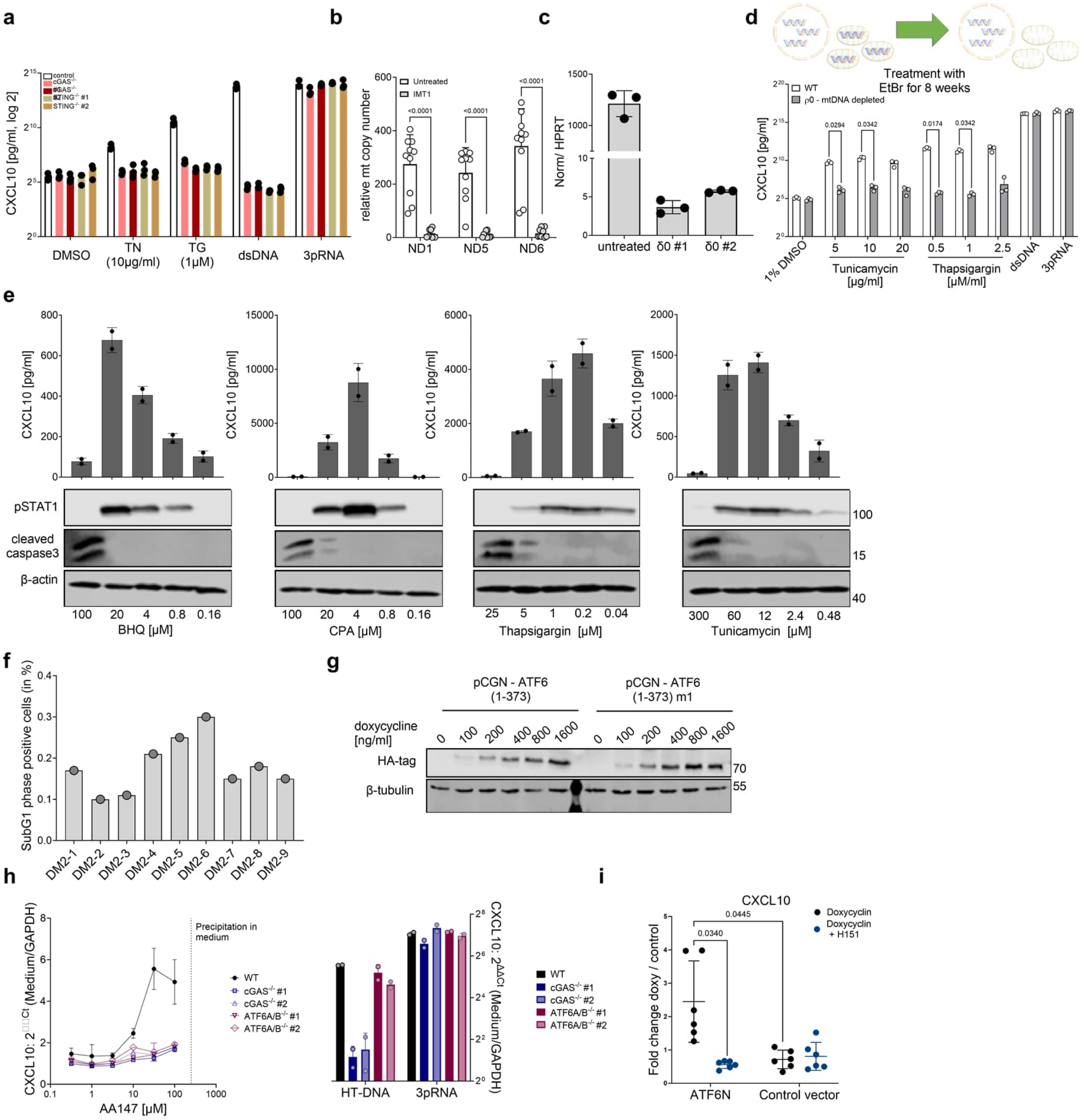

Supplementary Figure 4: ATF6N activation involved in ISG upregulation. a, CXCL10 expression in cGAS and STING deficient HT-29 cells after stimulation with tunicamycin (TN) and thapsigargin (TG). **b**, depletion of mtDNA in THP1 cells after treatment with IMT1 for 14 days. **c**, **d**, THP-1 cells were treated with EtBr for 8 weeks to deplete mitochondrial DNA. (**c**) ND1 levels after EtBr treatment. (**d**) CXCL10 protein levels by ELISA from EtBr-treated THP-1 cells after treatment with TN, TG, the cGAS agonist herring testis (HT)-DNA or the RIG-I agonist 3pRNA. **e**, CXCL10 protein expression using ELISA and protein expression of pSTAT1 and cleaved caspase-3 by immunoblotting after stimulation with four ER stress inducers. **f**, amount of subG1 positive cells in patient fibroblasts was analyzed by flow cytometry after propidium iodide staining. **g**, THP1 cells were transduced with a doxycycline-inducible ATF6N or mutated ATF6N control vector. **h**, THP-1 cells of the indicated genotype were stimulated with the ATF6N activator AA147 at the indicated concentrations or with HT-DNA or 3pRNA. CXCL10 mRNA induction was determined using RT-PCR. **i**, CXCL10 mRNA levels after induction of ATF6N with or without pre-treatment with the STING inhibitor H151. Data are shown as mean ± SD. a-i include data from one (f) two (e, h), three (a, c, d), six (i) or ten (b) independent experiments. g, representative for 3 independent experiments. Statistical

significance was assessed using multiple-paired t-test and the Holm-Sidak post hoc test (b, d) and two-way ANOVA and the Bonferroni post hoc test (i).

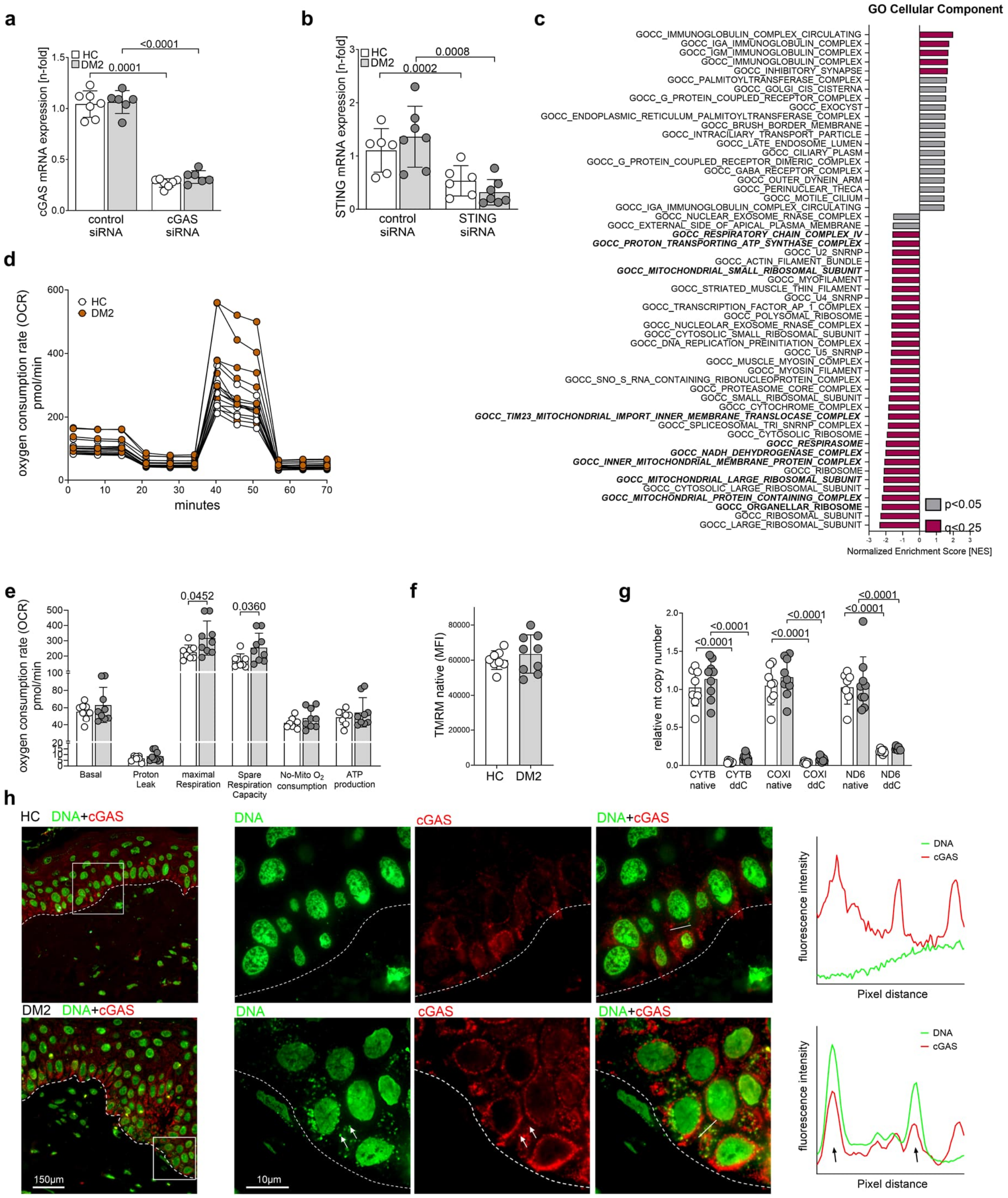

Supplementary Figure 5: Analysis of mitochondrial function in patients with myotonic dystrophy type 2. mRNA expression of cGAS and STING was detected by RT-PCR after siRNA knockdown of cGAS (**a**, DM2 n=6, HC n=7) or STING (**b**, DM2 n=7, HC n=6). **c**, *Gene Set Enrichment Analysis* using the GSEA software. The utilized gene sets are derived from the GO *Cellular Component* Ontology as provided by the *Molecular Signatures Database* (MSigDB). Pathways associated with mitochondria are shown in bold. **d**, **e**, measurement of oxygen consumption rate (OCR) in fibroblasts from n=9 DM2 patients and n=8 controls. One representative experiment of three is shown. **f**, fibroblasts of n=9 DM2 patients and n=8 controls were stained with tetramethylrhodamin-methylester (TMRM) and analyzed by flow cytometry. Mean fluorescence intensity (MFI) is shown. **g**, depletion of mtDNA in fibroblasts after treatment with 2',3' dideoxycytidine (ddC) for 9 days in n=8 HC and n=9 DM2 patients. **h**,

immunohistochemistry (IHC) showing DNA (green) and cGAS (red) immunostaining in a skin section from a healthy control and a DM2 patient. Overlap of cGAS and DNA in the cytoplasm (DM2 patient) was detected by Fiji *Plot Profile* analysis. Data are shown as mean ± SD. a, b, f, g, include data from two (g, h), three (f), six (b) or ten (a) independent experiments. d, e show one representative experiment out of three. Statistical significance was assessed using student's t-test (e), paired student's t-test (a, b, g) or Mann-Whitney U test (e).

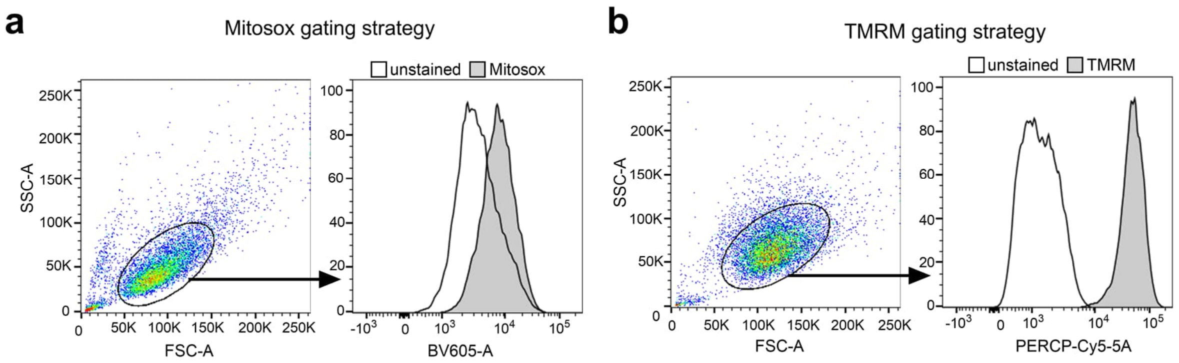

Supplementary Figure 6: Gating strategies for the fluorogenic dyes Mitosox (a) and TMRM (b).

Supplementary Tables

Supplementary Table 1: Prevalence of autoimmune diseases in DM2 and DM1 patients

Characteristic	DM2	DM1
**n**	37	13
**Female sex (n (%))**	26 (70.3%)	6 (46.2%)
**Age (years) mean**	56.1	48
**Autoimmune diseases**		
Alopecia areata (n)	2	0
Connective tissue diseases:		
MCTD (n)	1	0
Morphea (n)	1	0
Eosinophilic fasciitis (n)	1	0
Systemic sclerosis (n)	1	0
Unspecific collagenosis (n)	1	0
Crohn's disease (n)	1	0
Hyperthyroidism (n)	1	0
Lichen planus (n)	1	0
Hypothyroidism (n)	0	1
Psoriasis vulgaris (n)	1	0
Rheumatoid arthritis (n)	2	0
Type 1 diabetes (n)	1	0
Vitiligo (n)	2	0
**Total no of patients with ≥ 1 autoimmune diseases (in %)**	15 (40.5%)	1 (7.7%)
**Positive ANA (n (%))**	28 (75.7%)	8 (61.5%)

Supplementary Table 2: Clinical and laboratory characteristics of DM2 patients.

Patient	Gender	Age at clinical visitation (years)	CCTG Repeats (n)	Positive autoantibodies	Autoimmune diseases	ALAT (µmol/ml*s) Norm range male: <0.85 Norm range female: <0.6	ASAT (µmol/ml*s) Norm range male: <0.85 Norm range female: <0.6	GGT (µmol/ml*s) Norm range male: <1.19 Norm range female: <0.7	CK (µg/l) Norm range male: <3.71 Norm range female: <2.78	Myoglobine (µg/l) Norm range male: <72 Norm range female: <58
1	female	50	1500	1:160	none	1.29	1.06	2.77	6.03	63.1
2	female	64	7500	1:10240	Systemic sclerosis	0.72	0.87	2.03	7.49	177.2
3	female	63	7500	1:160	Rheumatoid arthritis	0.32	0.56	0.24	5.26	183.1
4	male	68	8000	1:1280	Hyperthyroidism	0.99	1.02	4.55	9.41	299.1
5	female	49	4000	no	Rheumatoid arthritis	0.46	0.65	1.29	4.02	77.5
6	female	65	*	no	none	0.52	0.72	3.77	4.13	105.7
7	male	32	4000	1:160	Alopecia areata	0.82	0.74	0.64	13.46	123.5
8	female	56	2000-3500	1:160	none	0.53	0.54	0.42	6.94	124.8
9	female	61	> 75	1:320	Alopecia areata	0.41	0.58	0.39	5.92	104.5
10	female	58	4000	no	Lichen planus	0.47	0.35	1.19	1.56	25.3
11	male	68	> 75	1:320	none	0.43	0.62	1.15	8.05	190.7
12	female	71	> 75	no	none	1.11	1.21	1.44	10.16	135.5
13	male	36	> 75	1:160	Type 1 Diabetes	0.35	0.48	0.34	6.31	75.5
14	male	51	<3750	no	none	0.66	0.49	2.82	2.02	40.6
15	male	50	4000	1:160	none	0.66	0.41	4.95	4.22	71.3
16	female	72	3500	1:320	none	0.5	0.54	0.47	9.13	116.4
17	male	61	5500	1:320	none	0.8	0.92	0.66	25.48	350.2
18	female	66	> 75	1:160	none	0.39	0.22	1.24	1.19	36.8
19	female	48	4000	1:160	none	0.38	0.57	0.39	7.48	75.6
20	male	28	3500	no	none	0.52	0.57	0.46	10.95	47.8
21	male	26	1000	1:160	none	0.52	1.32	0.59	28.81	68.9
22	female	28	4000	no	none	0.4	0.35	0.47	2.33	23.7
23	female	51	4000	no	none	0.2	0.31	0.42	1.2	30.5
24	female	73	> 75	1:640	Vitiligo	0.55	0.73	0.9	5.74	175
25	male	53	2000	1:320	Crohn's disease	0.98	0.67	1.17	8.34	141.7
26	female	76	2000	1:640	MCTD	0.6	0.78	0.92	5.19	90.5
27	female	57	4000	1:320	none	0.34	0.4	0.61	1.73	36.2
28	female	62	8000	1:160	none	0.89	0.65	4.59	9.5	176.9
29	female	61	>7500	1:320	none	0.59	0.59	0.74	4.1	65.5
30	female	68	> 75	1:160	none	0.33	0.57	1.15	3.82	59.2
31	female	73	4000	1:160	none	0.66	0.7	2.11	8.69	132.5
32	female	46	> 75	1:160	Eosinophilic fasciitis	0.76	0.56	1.13	5.71	72.5
33	female	33	> 75	no	Psoriasis vulgaris	0.68	0.37	0.24	3.83	44.7
34	male	64	> 75	1:160	none	0.34	0.83	3.34	1.42	-
35	female	68	1000	1:640	Vitiligo, Morphea	0.41	0.36	0.36	3.24	105.6
36	female	62	40	1:320	Unspecific collagenosis	1.2	1	3.71	4.55	46.6
37	female	58	7500	1:160	none	0.87	0.77	0.95	3.26	225.3
			* positive family history							

Supplementary Table 3: Disease specific antibodies

Characteristic	DM2
**Positive autoantibodies**	
**n**	37
ANA (n (%))	28 (75.7 %)
ENA (n (%))	5 (13.5 %)
SSA (n (%))	3 (8.1%)
SS/RNP (n (%))	1 (2.7%)
Centromer (n (%))	1 (2.7%)
AMA (n (%))	2 (5.4 %)

Supplementary Table 4: Genotypes of gene-edited THP-1 cell lines

Clone	Gene	Target sequence	Allele1 Indel	Allele1 sequence	Allele2 Indel	Allele2 sequence	Allele3 Indel	Allele3 sequence
cGAS #1	CGAS	GGCCGCCCGTCCGCGCAACT	+1bp	CCCAGT[+T]TGCGCG				
cGAS #2	CGAS	GGCCGCCCGTCCGCGCAACT	-1bp	CCCAGT.GCGCGG	-49bp	AGGCCG[+GCGGGTCTCGACCCCCGTTCGCCTAGG]...GCGCCC		
cGAS #3	CGAS	GGCCGCCCGTCCGCGCAACT	-1bp	CCCAGT.GCGCGG	+1bp	CCCAGT[+T]TGCGCG		
STING #1	STING1	CTAGCCCCCAAAGGGTCACC	+1bp	AGGGTC[+A]ACCAGG	+1bp	AGGGTC[+T]ACCAGG		
STING #2	STING1	CTAGCCCCCAAAGGGTCACC	+1bp	AGGGTC[+C]ACCAGG	+1bp	AGGGTC[+G]ACCAGG		
STING #3	STING1	CTAGCCCCCAAAGGGTCACC	-1bp	CCCAGT.GCGCGG	+1bp	CCCAGT[+T]TGCGCG		
IRF3 #1	IRF3	GCACGCGCTTCCGCATCCCT	-1bp	CCAAGG.ATGCGG				
IRF3 #2	IRF3	GCACGCGCTTCCGCATCCCT	-7bp	CCAAGG...AAGCGC				
IRF3 #3	IRF3	GCACGCGCTTCCGCATCCCT	-38bp	GAAATC...GGATGC	-1bp	CCAAGG.ATGCGG		
EIF2AK3 #1	EIF2AK3	TCGTCTGGTTCCGGACCCCG	-1bp	CCTCGG.GTCCGG	+1bp	CCTCGG[+G]GGTCCG		
EIF2AK3 #2	EIF2AK3	TCGTCTGGTTCCGGACCCCG	+1bp	CCTCGG[+G]GGTCCG				
EIF2AK3 #3	EIF2AK3	TTTCCCATCCTTAAGTAACC	-7 bp	TTAAGT...CAGATG	-10bp	CTTAAG...GATGCA		
ERN1 #1	ERN1	GTGGAAGTACCCGTTCCCCA	-1bp	CCTTGG.GAACGG				
ERN1 #2	ERN1	GTGGAAGTACCCGTTCCCCA	-1bp	CCTTGG.GAACGG				
ERN1 #3	ERN1	GTGGAAGTACCCGTTCCCCA	-1bp	CCTTGG.GAACGG	-7bp	CCTTGG...GTACTT		
ATF6A/B #1	ATF6A	TGAAAGAGTCCCGGGCTAAA	+1bp	CCTTTT[+A]AGCCCG	-11bp	CCTTTTA...TTTCAC		
	ATF6B	GACGCAGCTCTTCCGTTGCC	-1bp	CCGGGC.ACGGAA	+1bp	CCGGGC[+G]AACGGA		
ATF6A/B #2	ATF6A	TGAAAGAGTCCCGGGCTAAA	-46bp	TGGGGG...ACTCTT	-2bp	CACCTT..AGCCCG		
	ATF6B	GACGCAGCTCTTCCGTTGCC	-5bp	TCCGGG...GAAGAG	-2bp	CCGGGC..CGGAAG	-1bp	CCGGGC.ACGGAA
ATF6A/B #3	ATF6A	TGAAAGAGTCCCGGGCTAAA	+1bp	CCTTTT[+A]AGCCCG	+1bp	CCTTTT[+G]AGCCCG	-1bp	ACCTTT.AGCCCG
	ATF6B	GACGCAGCTCTTCCGTTGCC	-1bp	CCGGGC.ACGGAA	+1bp	CCGGGC[+G]AACGGA		

Insertions are annotated as [+N]. Deletions are indicated with dots.
In case of combined insertion/deletion, net indel size is given.
No sequence for allele 2 means identical to allele 1 (homozygous).
cGAS #1+2 and STING #1+2 have been published previously (https://pubmed.ncbi.nlm.nih.gov/34381047/).
ATF6A/B clones were generated by consecutive targeting of ATF6B (yielding 2 parental clones) and then A.

Supplementary Table 5: Primer sequence

Name	sequence (5‘ > 3‘)	Company
HPRT	Fw: TGGAAAGGGTGTTTATTCCTCAT<br>Rv: ATGTAATCCAGCAGGTCAGCAA	Eurofins
IFIT1	FW: AGAAGCAGGCAATCACAGAAAA<br>RV: CTGAAACCGACCATAGTGGAAAT	Eurofins
IFI44	FW: CTGGGGCTGAGTGAGAAAGA<br>RV: AGCGATGGGGAATCAATGTA	Eurofins
IFI44L	FW: AGCCGTCAGGGATGTACTATAAC<br>RV: AGGGAATCATTTGGCTCTGTAGA	Eurofins
CXCL10	FW: GGTGAGAAGAGATGTTCGAATCC<br>RV: GTCCATCCTTGGAAGCACTGCA	Eurofins
ISG15	FW: GAGAGGCAGCGAACTCATCT<br>RV: CTTCAGCTCTGACACCGACA	Eurofins
IFI27	FW: TGCTCTCACCTCATCAGCAGT<br>RV: CACAACTCCTCCAATCACAACT	Eurofins
Viperin	FW: CCAGTGCAACTACAAATGCGGC<br>RV: CGGTCTTGAAGAAATGGCTCTCC	Eurofins
IFI16	FW: ACTGAGTACAACAAAGCCATTTGA<br>RV: TTGTGACATTGTCCTGTCCCCAC	Eurofins
IRF7	FW: TACCATCTACCTGGGCTTCG<br>RV: GCTCCATAAGGAAGCACTCG	Eurofins
TLR3	FW: TCCCAGCCTTACAGAGAAGC<br>RV: CCTGTGAGTTCTTGCCCAAT	Eurofins
CNBP	FW: GGAGCCCAAGAGAGAGCGA<br>RV: TGGCTACATGACCAGTTTCAC	Eurofins
DHX36	FW: CCCACCATCAAATGAGGCAGTG<br>RV: TGTGGCTCAACGGGTAATCGTG	Eurofins
DDX58	FW: GTGCAAAGCCTTGGCATGT<br>RV: TGGCTTGGGATGTGCTCTACTC	Eurofins
MDA5	FW: GTTGAAAAGGCTGGCTGAAAAC<br>RV: TCGATAACTCCTGAACCACTG	Eurofins
MAVS	FW: ATAAGTCCTGAGGGCACCTTT<br>RV: GTGACTACCAGCACCCCTGT	Eurofins
BiP	FW: TGTTCAACCAATTATCAGCAAACTC<br>RV: TTCTGCTGTATCCTCTTCACCAGT	Eurofins
XBP1 unspliced	FW: GCAGCACTCAGACTACGTGCAC<br>RV: GCTGGCAGGCTCTGGGGAAG	Eurofins
XBP1 spliced	FW: TGCTGAGTCCGCAGCAGGTG<br>RV: GCTGGCAGGCTCTGGGGAAG	Eurofins
PERK	FW: ATCCCCCATGGAACGACCTG<br>RV: ACCCGCCAGGGACAAAAATG	Eurofins
Mx1	FW: TTCGGCTGTTTACCAGACTCC<br>RV: CAAAGCCTGGCAGCTCTCTAC	Eurofins
ATF6	FW: AGCAGCACCCAAGACTCAAAC<br>RV: GCATAAGCGTTGGTACTGTCTGA	Eurofins
cGAS	FW:GGGAGCCCTGCTGTAACACTTCTTAT<br>RV: CCTTTGCATGCTTGGGTACAAGGT	Eurofins
STING	FW: CCAGAGCACACTCTCCGGTA<br>RV: CGCATTTGGGAGGGAGTAGTA	Eurofins
IFNb	FW: GACATCCCTGAGGAGATTAAGCA<br>RV: CTGGAGCATCTCATAGATGGTCAA	Eurofins
ND1	FW: GGCATTCCTAATGCTTACCG<br>RV: CGTCAGCGAAGGGTTGTAGT	Eurofins
COXI	FW: TTCGCCGACCGTTGACTATTCTCT<br>RV: AAGATTATTACAAATGCATGGGC	Eurofins
ND6	FW: GGGGTCAGGGGTTGAGGTC<br>RV: GTTTACCACAACCACCACCCC	Eurofins
CytB	FW: AGACAGTCCCACCCTCACAC	Eurofins

	RV: GGTGATTCCTAGGGGGTTGT	
ACTB	FW: TCACCCACACTGTGCCCATCTACGA<br>RV: CAGCGGAACCGCTCATTGCCAATGG	Eurofins
DDX58	FW: CTGCTCTGCAGAAAGTGCAAA<br>RV: GGCTTGGGATGTGGTCTACT	Eurofins
CXCL10	FW: CCACGTGTTGAGATCATTGCT<br>RV: TGCATCGATTTTGCTCCCCT	Eurofins
ND5	FW: ATTTTATTTCTCCAACATACTCGGATT<br>RV: GGGCAGGTTTTGGCTCGTA	Eurofins
ND6	FW: CCAATAGGATCCTCCCGAAT<br>RV: AGGTAGGATTGGTGCTGTGG	Eurofins
IFNb	FW: ACGCCGCATTGACCATCTAT<br>RV: GTCTCATTCCAGCCAGTGCT	Eurofins
ActB	FW: CACAGAGCCTCGCCTTTGC<br>RV: AATCCTTCTGACCCATGCCC	Eurofins

Supplementary References:

1. Kirou, K.A., *et al.* Coordinate overexpression of interferon-alpha-induced genes in systemic lupus erythematosus. *Arthritis Rheum* 50, 3958-3967 (2004).
