# Supplementary material for: Chronic endoplasmic reticulum stress in myotonic dystrophy type 2 promotes autoimmunity via mitochondrial DNA release
Source: Nat Commun. 2024 Feb 20;15:1534. doi: 10.1038/s41467-024-45535-1 (PMC10879130; doi:10.1038/s41467-024-45535-1)
Supplement: Supplementary file 2 — Reporting Summary [file 41467_2024_45535_MOESM2_ESM.pdf]

nature portfolio

Corresponding author(s): Claudia Günther

Last updated by author(s): 2024/01/08

Reporting Summary

Nature Portfolio wishes to improve the reproducibility of the work that we publish. This form provides structure for consistency and transparency in reporting. For further information on Nature Portfolio policies, see our Editorial Policies and the Editorial Policy Checklist.

Statistics

For all statistical analyses, confirm that the following items are present in the figure legend, table legend, main text, or Methods section.

n/a	Confirmed	
☐	☒	The exact sample size (*n*) for each experimental group/condition, given as a discrete number and unit of measurement
☐	☒	A statement on whether measurements were taken from distinct samples or whether the same sample was measured repeatedly
☐	☒	The statistical test(s) used AND whether they are one- or two-sided<br>*Only common tests should be described solely by name; describe more complex techniques in the Methods section.*
☐	☒	A description of all covariates tested
☐	☒	A description of any assumptions or corrections, such as tests of normality and adjustment for multiple comparisons
☐	☒	A full description of the statistical parameters including central tendency (e.g. means) or other basic estimates (e.g. regression coefficient) AND variation (e.g. standard deviation) or associated estimates of uncertainty (e.g. confidence intervals)
☐	☒	For null hypothesis testing, the test statistic (e.g. *F*, *t*, *r*) with confidence intervals, effect sizes, degrees of freedom and *P* value noted<br>*Give P values as exact values whenever suitable.*
☒	☐	For Bayesian analysis, information on the choice of priors and Markov chain Monte Carlo settings
☒	☐	For hierarchical and complex designs, identification of the appropriate level for tests and full reporting of outcomes
☒	☐	Estimates of effect sizes (e.g. Cohen's *d*, Pearson's *r*), indicating how they were calculated

Our web collection on statistics for biologists contains articles on many of the points above.

Software and code

Policy information about availability of computer code

Data collection	Wave Software 26.3; Zeiss Zen Blue 3.1; ImageQuant LAS 4000 1.2; BDFACSDiva Software v9.0.1; Bio-Rad CFX Maestro 2.2 (5.2.008.0222); Omega Software Version 5.70; i-control Software Versions-Nr. 2.0.10; Magellan Software Version 7.2.1.6
Data analysis	Graphpad Prism 10.0.3 (275); Wave Software 2.6.3; Cellprofiler 3.1.8; Arivis Vision 4D 3.5.1; FlowJo_v10.8.1; Rstudio R 4.2.1 packages heatmap.2; Image Quant TL 8.1.0.0; fastqc (https://www.bioinformatics.babraham.ac.uk/projects/fastqc/); trimmomatic (https://academic.oup.com/bioinformatics/article/30/15/2114/2390096?login=true); STAR(https://academic.oup.com/bioinformatics/article/29/1/15/272537?login=true); featureCounts (https://academic.oup.com/bioinformatics/article/30/7/923/232889); DESeq2 (https://genomebiology.biomedcentral.com/articles/10.1186/s13059-014-0550-8); Omega MARS Version 3.42 R5; GIMP 2.10.36; Microsoft Office 2016; ImageJ 1.53n Java 1.8.0_172 (64-bit); ImageStudio Lite Version 5.2.5 LI-COR BIOSCIENCES

For manuscripts utilizing custom algorithms or software that are central to the research but not yet described in published literature, software must be made available to editors and reviewers. We strongly encourage code deposition in a community repository (e.g. GitHub). See the Nature Portfolio guidelines for submitting code & software for further information.

Data

Policy information about availability of data

All manuscripts must include a data availability statement. This statement should provide the following information, where applicable:

- Accession codes, unique identifiers, or web links for publicly available datasets
- A description of any restrictions on data availability
- For clinical datasets or third party data, please ensure that the statement adheres to our policy

The RNA-Sequencing data have been deposited in Gene Expression Omnibus (GEO) database under accession code GSE242388 (https://www.ncbi.nlm.nih.gov/geo/query/acc.cgi?acc=GSE242388). The data generated in this study are provided in the Source Data file.

Research involving human participants, their data, or biological material

Policy information about studies with human participants or human data. See also policy information about sex, gender (identity/presentation), and sexual orientation and race, ethnicity and racism.

Reporting on sex and gender	We included 6 male and 11 female. Specific information on gender has not been collected.
Reporting on race, ethnicity, or other socially relevant groupings	All patients were white
Population characteristics	Patients with mytonic dystrophy type 2 (age range: 26 years - 76 years); patients with myotonic dystrophy type 1 (age range: 20 years - 75 years); healthy controls (age range: 28 years - 85 years)
Recruitment	Patients were enrolled at the department of Neurology and Dermatology at university hospital Carl Gustav Carus Dresden, when they were seen for routine medical care.
Ethics oversight	Ethikkommission an der Technischen Universitat Dresden

Note that full information on the approval of the study protocol must also be provided in the manuscript.

Field-specific reporting

Please select the one below that is the best fit for your research. If you are not sure, read the appropriate sections before making your selection.

☒ Life sciences ☐ Behavioural & social sciences ☐ Ecological, evolutionary & environmental sciences

For a reference copy of the document with all sections, see nature.com/documents/nr-reporting-summary-flat.pdf

Life sciences study design

All studies must disclose on these points even when the disclosure is negative.

Sample size	Sample size depended on the enrollment of patients at the university hospital Carl Gustav Carus Dresden
Data exclusions	Outliers mainly caused by technical variation and where therefore excluded if they fullfill the Graphpad's online outlier test.
Replication	Number of replicats of every experiment can be found in the source data file.
Randomization	Randomization was restricted to the fact whether the disease was present or not.
Blinding	Partial blinding by allocation of IDs to patient samples.

Behavioural & social sciences study design

All studies must disclose on these points even when the disclosure is negative.

Study description	
Research sample	
Sampling strategy	

Data collection

Timing

Data exclusions

Non-participation

Randomization

Ecological, evolutionary & environmental sciences study design

All studies must disclose on these points even when the disclosure is negative.

Study description

Research sample

Sampling strategy

Data collection

Timing and spatial scale

Data exclusions

Reproducibility

Randomization

Blinding

Did the study involve field work? ☐ Yes ☐ No

Field work, collection and transport

Field conditions

Location

Access & import/export

Disturbance

Reporting for specific materials, systems and methods

We require information from authors about some types of materials, experimental systems and methods used in many studies. Here, indicate whether each material, system or method listed is relevant to your study. If you are not sure if a list item applies to your research, read the appropriate section before selecting a response.

Materials & experimental systems

n/a	Involved in the study	
☐	☒	Antibodies
☐	☒	Eukaryotic cell lines
☒	☐	Palaeontology and archaeology
☒	☐	Animals and other organisms
☒	☐	Clinical data
☒	☐	Dual use research of concern
☒	☐	Plants

Methods

n/a	Involved in the study	
☒	☐	ChIP-seq
☐	☒	Flow cytometry
☒	☐	MRI-based neuroimaging

Antibodies

Antibodies used

PKR, Cell Signaling, D7F7, #12297, 3; phospho-STAT1, Cell Signaling, 58D6, #9167, 18; cleaved caspase 3, Cell signaling, Asp175 #9661, 47; pPKR, Abcam, E120, ab32036, GR255462-31; LPAC, Merck, polyclonal, ABN2258, O.3222902; PERK, Cell signaling, D11A8, #5683, 6; ATF6, Cell signaling, D4Z8V, #65880, 3; ATF6-N, Novus Biologicals, polyclonal, NBP1-75478, B-1; IRE1 , Cell signaling, 14C10, #3294, 12; eIF2 , Cell signaling, polyclonal, #9722, 15; peIF2 , Cell signaling, D9G8, 3398T, 6; CNBP, Sigma, polyclonal, SAB2100453, QC28860; DHX36, santa cruz, B-6, sc-377485, G1816; GAPDH, Cell Signaling, 14C10, #2118, 14; b-actin, Cell Signaling, 13E5, #4970, 18; a-Tubulin, Neomarker, DM1A, MS-581-P1, 581P 2005F; anti DNA, Progen, AC-30-10, #61014, 707031; cGAS, Novus Biologicals, polyclonal, NBP1-86761, 1117415; Anti-HA directly conjugated, Thermo Fisher Scientific, PA1-29751, RL2303931B; b-actin, Licor, 926-42212, D30303-03; IRDye 680RD goat anti-mouse IgG, Licor, 926-68070, C70908-04,D30207-05; Anti beta-actin-HRP, SCBT:beta Actin (C4) sc-47778, D1916; Anti-rabbit IgG Peroxidase antibody produced in goat, Sigma A0545, #0000103434, #0000118493; ECLTM Anti-mouse IgG, Horseradish Peroxidase linked whole antibody (from sheep), Cytiva NA931V, 17415554; QAGR, Merck ABN2271, Q3222900; goat anti-mouse IgM AF488, LifeTechnologies #A-21042, 1964383; goat anti-rabbit IgG-AF546 LifeTechnologies, #A-11071, 1896381; BG4, provided by Katrin Paeschke self-made; MxA provided by O. Haller self-made;

Validation

PKR: validated by manufacturer, According to manufacturer, cited in 29 publications (28 December 2023) for immunoblotting of PKR https://www.cellsignal.com/products/primary-antibodies/pkr-d7f7-rabbit-mab/12297; pSTAT1: validated by manufacturer, According to manufacturer, cited in 81 publications (16 December 2023) for immunoblotting of cleaved human phospho-STAT1 https://www.cellsignal.com/products/primary-antibodies/phospho-stat1-tyr701-58d6-rabbit-mab/9167; cleaved caspase 3: validated by manufacturer, According to manufacturer, cited in 631 publications (16 December 2023) for immunoblotting of cleaved human caspase-3 https://www.cellsignal.com/products/primary-antibodies/cleaved-caspase-3-asp175-antibody/9661; pPKR: validated by manufacturer, According to manufacturer, cited in 107 publications (28 December 2023). https://www.abcam.com/products/primary-antibodies/pkr-phospho-t446-antibody-e120-ab32036.html; LPAC and QAGR: Cited in Banez-Coronel, M., et. al. (2015). Neuron. 88(4):667-77 https://www.merckmillipore.com/DE/de/product/Anti-poly-LPAC,MM_NF-ABN2258-100UL; PERK: validated by manufacturer, According to manufacturer, cited in 155 publications (28 December 2023) for immunoblotting of PERK. https://www.cellsignal.com/products/primary-antibodies/perk-d11a8-rabbit-mab/5683; ATF6: validated by manufacturer, According to manufacturer, cited in 76 publications (28 December 2023) for immunoblotting of ATF6. https://www.cellsignal.com/products/primary-antibodies/atf-6-d4z8v-rabbit-mab/65880; ATF6-N: validated by manufacturer According to manufacturer, cited in 4 publications (28 December 2023) for immunoblotting of ATF6. https://www.novusbio.com/products/atf6-antibody_nbp1-75478#reviews-publications and our lab (Supplementary Fig. 3F); IRE1a: validated by manufacturer, According to manufacturer, cited in 388 publications (28 December 2023) for immunoblotting of IRE1a. https://www.cellsignal.com/products/primary-antibodies/ire1a-14c10-rabbit-mab/3294; eIf2a: validated by manufacturer, According to manufacturer, cited in 472 publications (28 December 2023) for immunoblotting of eIf2a. https://www.cellsignal.com/products/primary-antibodies/eif2a-antibody/9722; peIF2a: validated by manufacturer. According to manufacturer, cited in 397 publications (28 December 2023) for immunoblotting of peIf2a. https://www.cellsignal.com/products/primary-antibodies/phospho-eif2a-ser51-d9g8-xp-rabbit-mab/3398; CNBP: validated by manufacturer. According to CiteAb, cited in 1 publications (28 December 2023) https://www.citeab.com/antibodies/2297229-sab2100453-anti-cnbp-antibody-produced-in-rabbit?des=5060b48424801a4c; DHX36: validated by manufacturer. According to manufacturer, cited in 1 publications (28 December 2023) for immunoblotting ofDHX36. https://www.scbt.com/p/ddx36-antibody-b-6; GAPDH: validated by manufacturer. According to manufacturer, cited in 4166 publications (28 December 2023) for immunoblotting of GAPDH. https://www.cellsignal.com/products/primary-antibodies/gapdh-14c10-rabbit-mab/2118; b-actin: validated by manufacturer. According to manufacturer, cited in 3283 publications (28 December 2023) for immunoblotting of b-actin. https://www.cellsignal.com/products/primary-antibodies/b-actin-13e5-rabbit-mab/4970; a-tubulin: validated by manufacturer http://tools.thermofisher.com/content/sfs/brochures/D11896~.pdf; anti-DNA: validated by manufacturer. According to manufacturer, cited in 12 publications (28 December 2023) for immunofluorescence staining of DNA. https://www.progen.com//anti-DNA-mouse-monoclonal-AC-30-10-lyophilized-purified/61014; cGAS: validated by manufacturer. According to manufacturer, cited in 1 publications (28 December 2023) for immunofluorescence staining of cGAS. https://www.novusbio.com/products/cgas-antibody_nbp1-86761#reviews-publications; Anti-HA: validated by manufacturer. Manufacturer's website: https://www.thermofisher.com/antibody/product/HA-Tag-Antibody-Polyclonal/PA1-29751 Also in 4 publications with immunoblotting https://www.citeab.com/antibodies/95856-pa1-29751-ha-tag-polyclonal-antibody-hrp?des=9fa26192d2b9c7e9; b-actin: not validated by manufacturer, used for this application in multiple publications. According to CiteAb, cited in 4 publications (16 December 2023) for immunoblotting of human B-actin https://www.citeab.com/antibodies/3432140-926-42212-actin-rabbit-monoclonal-antibody?des=20489c1182c146c1; IRDye 680RD: validated by manufacturer. Licor: https://www.licor.com/bio/reagents/irdye-680rd-goat-anti-mouse-igg-secondary-antibody, According to CiteAb, cited in 1261 publications (16 December 2023) https://www.citeab.com/antibodies/2872729-926-68070-irdye-680rd-goat-anti-mouse-igg-h-l; beta-actin-HPR: validated by manufacturer. https://www.scbt.com/p/beta-actin-antibody-c4, According to CiteAb, cited in 1044 publications (16 December 2023) for immunoblotting of human B-actin https://www.citeab.com/antibodies/788577-sc-47778-beta-actin-antibody-c4?des=09496434d18b0586; Anti-rabbit IgG Peroxidase: validated by manufacturer. According to CiteAb, cited in 970 publications (28 December 2023) for immunoblotting. https://www.citeab.com/antibodies/1523796-a0545-anti-rabbit-igg-whole-molecule-peroxidase-an?des=2548aa57149dcf8f; ECLTM anti-mouse IgG Peroxidase: validated by maunfacturer. According to CiteAb, cited in 73 publications (28 December 2023) for immunoblotting. https://www.citeab.com/antibodies/3288287-na931-1ml-amersham-ecl-mouse-igg-hrp-linked-whole-a; goat anti-mouse IgM AF488: validated by manufacturer. According to manufacturer, cited in 443 publications (28 December 2023). https://www.thermofisher.com/antibody/product/Goat-anti-Mouse-IgM-Heavy-chain-Cross-Adsorbed-Secondary-Antibody-Polyclonal/A-21042; goat anti-rabbit IgG AF546: validated by manufacturer. According to manufacturer, cited in 122 publications (28 December 2023). https://www.thermofisher.com/antibody/product/Goat-anti-Rabbit-IgG-H-L-Cross-Adsorbed-Secondary-Antibody-Polyclonal/A-11071

Eukaryotic cell lines

Policy information about cell lines and Sex and Gender in Research

Cell line source(s)	primary cell Source: Universitaetsklinikum Carl Gustav Carus; sex: HC 1 f, HC 2 m, HC 3 f, HC 4 f, HC 5 m, HC 6 m, HC 7 f, HC 8 f; DM2_1 f, DM2_2 f, DM2_3 f, DM2_4 m, DM2_5 f, DM2_6 f, DM2_7 f, DM2_8 m, DM2_9 m;<br>THP1 Source: commercial<br>HT-229 cells: provided by Katarzyna Andryka
Authentication	not relevant for primary cells. THP1 and HT-29 cells were validated using STR profiling from Eurofins
Mycoplasma contamination	We confirm that all cell lines tested negative for mycoplasma
Commonly misidentified lines<br>(See ICLAC register)	HT-29 cells validated by STR profiling

Palaeontology and Archaeology

Specimen provenance	
Specimen deposition	
Dating methods	

☐ Tick this box to confirm that the raw and calibrated dates are available in the paper or in Supplementary Information.

Ethics oversight	

Note that full information on the approval of the study protocol must also be provided in the manuscript.

Animals and other research organisms

Policy information about studies involving animals; ARRIVE guidelines recommended for reporting animal research, and Sex and Gender in Research

Laboratory animals	
Wild animals	
Reporting on sex	
Field-collected samples	
Ethics oversight	

Note that full information on the approval of the study protocol must also be provided in the manuscript.

Clinical data

Policy information about clinical studies

All manuscripts should comply with the ICMJE guidelines for publication of clinical research and a completed CONSORT checklist must be included with all submissions.

Clinical trial registration	
Study protocol	

Data collection

Outcomes

Dual use research of concern

Policy information about dual use research of concern

Hazards

Could the accidental, deliberate or reckless misuse of agents or technologies generated in the work, or the application of information presented in the manuscript, pose a threat to:

No	Yes	
☒	☐	Public health
☒	☐	National security
☒	☐	Crops and/or livestock
☒	☐	Ecosystems
☒	☐	Any other significant area

Experiments of concern

Does the work involve any of these experiments of concern:

No	Yes	
☒	☐	Demonstrate how to render a vaccine ineffective
☒	☐	Confer resistance to therapeutically useful antibiotics or antiviral agents
☒	☐	Enhance the virulence of a pathogen or render a nonpathogen virulent
☒	☐	Increase transmissibility of a pathogen
☒	☐	Alter the host range of a pathogen
☒	☐	Enable evasion of diagnostic/detection modalities
☒	☐	Enable the weaponization of a biological agent or toxin
☒	☐	Any other potentially harmful combination of experiments and agents

Plants

Seed stocks

Novel plant genotypes

Authentication

ChIP-seq

Data deposition

☐ Confirm that both raw and final processed data have been deposited in a public database such as GEO.

☐ Confirm that you have deposited or provided access to graph files (e.g. BED files) for the called peaks.

Data access links
May remain private before publication.

Files in database submission

Genome browser session
(e.g. UCSC)

Methodology

Replicates

Sequencing depth

Antibodies

Peak calling parameters

Data quality

Software

Flow Cytometry

Plots

Confirm that:

- [ ] The axis labels state the marker and fluorochrome used (e.g. CD4-FITC).
- [ ] The axis scales are clearly visible. Include numbers along axes only for bottom left plot of group (a 'group' is an analysis of identical markers).
- [ ] All plots are contour plots with outliers or pseudocolor plots.
- [ ] A numerical value for number of cells or percentage (with statistics) is provided.

Methodology

Sample preparation	To detect mitochondrial stress, 100.000 fibroblasts were incubated for 30 minutes at 37 °C with MitoSOXTM (5 µM) and Image-iTTM TMRM Reagent (100 nM). After incubation, the staining was analyzed by flow cytometry.
Instrument	FACS Canto II, LSR II
Software	Flowlo_v10.8.1
Cell population abundance	Cell viability was approx. 96%
Gating strategy	Dead cells were excluded by PI staining. Excitation wavelength range: MitoSox: 396/610 nm; TMRM: 548/574

- [x] Tick this box to confirm that a figure exemplifying the gating strategy is provided in the Supplementary Information.

Magnetic resonance imaging

Experimental design

Design type

Design specifications

Behavioral performance measures

Acquisition

Imaging type(s)

Field strength

Sequence & imaging parameters

Area of acquisition

Diffusion MRI [ ] Used [ ] Not used

Preprocessing

Preprocessing software

Normalization

Normalization template

Noise and artifact removal

Volume censoring

Statistical modeling & inference

Model type and settings

Effect(s) tested

Specify type of analysis: ☐ Whole brain ☐ ROI-based ☐ Both

Statistic type for inference

(See Eklund et al. 2016)

Correction

Models & analysis

n/a	Involved in the study
☐	☐ Functional and/or effective connectivity
☐	☐ Graph analysis
☐	☐ Multivariate modeling or predictive analysis

Functional and/or effective connectivity

Graph analysis

Multivariate modeling and predictive analysis
