# Supplementary material for: Chronic endoplasmic reticulum stress in myotonic dystrophy type 2 promotes autoimmunity via mitochondrial DNA release
Source: Nat Commun. 2024 Feb 20;15:1534. doi: 10.1038/s41467-024-45535-1 (PMC10879130; doi:10.1038/s41467-024-45535-1)
Supplement: Supplementary file 3 — Description of Additional Supplementary Files [file 41467_2024_45535_MOESM3_ESM.docx]

**Description of Additional Supplementary Files**

File Name: Supplementary Data 1

Description: This dataset contains the 313 significantly upregulated genes identified by RNA sequencing in fibroblasts of 7 DM2 patients compared to 5 healthy controls.
